# A phenomenological computational model of the evoked action potential fitted to human cochlear implant responses

**DOI:** 10.1371/journal.pcbi.1010134

**Published:** 2022-05-27

**Authors:** Ángel Ramos-de-Miguel, José M. Escobar, David Greiner, Domingo Benítez, Eduardo Rodríguez, Albert Oliver, Marcos Hernández, Ángel Ramos-Macías

**Affiliations:** 1 University Institute of Intelligent Systems and Numerical Applications in Engineering (SIANI), University of Las Palmas de Gran Canaria, Las Palmas, Spain; 2 Department of Otolaryngology, Head and Neck Surgery, Complejo Hospitalario Universitario Insular Materno Infantil de Gran Canaria, Las Palmas, Spain; Medical University Hannover, GERMANY

## Abstract

There is a growing interest in biomedical engineering in developing procedures that provide accurate simulations of the neural response to electrical stimulus produced by implants. Moreover, recent research focuses on models that take into account individual patient characteristics.

We present a phenomenological computational model that is customized with the patient’s data provided by the electrically evoked compound action potential (ECAP) for simulating the neural response to electrical stimulus produced by the electrodes of cochlear implants (CIs). The model links the input currents of the electrodes to the simulated ECAP.

Potentials and currents are calculated by solving the quasi-static approximation of the Maxwell equations with the finite element method (FEM). In ECAPs recording, an active electrode generates a current that elicits action potentials in the surrounding auditory nerve fibers (ANFs). The sum of these action potentials is registered by other nearby electrode. Our computational model emulates this phenomenon introducing a set of line current sources replacing the ANFs by a set of virtual neurons (VNs). To fit the ECAP amplitudes we assign a suitable weight to each VN related with the probability of an ANF to be excited. This probability is expressed by a cumulative beta distribution parameterized by two shape parameters that are calculated by means of a differential evolution algorithm (DE). Being the weights function of the current density, any change in the design of the CI affecting the current density produces changes in the weights and, therefore, in the simulated ECAP, which confers to our model a predictive capacity.

The results of the validation with ECAP data from two patients are presented, achieving a satisfactory fit of the experimental data with those provided by the proposed computational model.

## Author summary

The cochlea, found in the inner ear, is the organ where the sound is transformed into an electrical pulse to be transmitted by the neurons to the auditory cortex. Hearing loss can be caused by damage to the hair cells, in which case neuronal excitation is impaired. CIs are devices that replace the normal function of the impaired/damaged Organ of Corti. Computational models allow a better understanding of the mechanisms involved in the electrical stimulation of the auditory nerve. These models can help biomedical engineers to develop new CIs with improved auditory performance. One important aspect of our model is its customization with the patient’s data provided by the recording of the evoked compound action potential (the synchronous firing of a population of electrically stimulated auditory nerve fibers). This phenomenological model allows us to predict the registers of neural stimulation produced when the auditory nerve is stimulated with the CIs. We have validated the proposed model with real data obtained from two patients with CIs.

## Introduction

The best-known and most successful implantable neurostimulator or neuroprosthesis is the CI, designed to provide a sense of sound to an adult or child with severe to profound hearing loss [1]. CI is composed of a microphone, a speech processor, a transmitter and receiver/stimulator, and an electrode array, which is inserted in the cochlea. A recent survey of the state-of-the-art of CIs could be found in [2].

In the past two decades, assessment of CI functionality is clinically recognized to be widely used through the electrically compound action potential (ECAP) (see for example [3]), which represents the synchronous firing of a population of electrically stimulated ANF. A clinical applicability of ECAPs is to obtain the threshold current to evoke acoustical perception and the maximum comfortable loudness levels before producing any kind of discomfort [4]. A recent overview of recording methodologies, response characteristics and potential applications of the ECAP is published in [5], highlighting the importance of this measure in CI clinical practice.

A crucially important aspect in neuronal modeling is to know the spread of excitation of nerve activity that is excited when it is subjected to neuronal stimulation produced by a CI. It is essential to predict the activation of nerve fibers out of the target region of each electrode of the CI (crosstalk) [6–8] and to design electrodes that improve the focalization [9–12]. Also, it could be useful to predict dead regions of the cochlea [13, 14]. Specifically, we want to know the relationship between the current applied by each electrode and the set of neurons of the auditory nerve that are excited.

The complexity of biological systems makes it impossible to build a deterministic model that provides a precise response to an external stimulus. As it is pointed in [15], “Most models of neural response to electrical stimulation, such as the Hodgkin–Huxley equations, are deterministic, despite significant physiological evidence for the existence of stochastic activity. For instance, the range of discharge probabilities measured in response to single electrical pulses cannot be explained at all by deterministic models.” As far as we know, the current computational cochlear models have a deterministic approach, that is, they try to accurately reproduce all the physical and biological processes involved in cochlear stimulation [6, 16–23]. This approach would be the appropriate if we knew precisely all the variables (conductivities of the media, patient-specific cochlear geometry, neuron positions in the nerve and its synapses positions of the electrodes, healthy state of the auditory nerve, possible fibrosis on the electrode, etc.) implicated in the problem. In addition, it would be needed to know the precise excitation mechanisms of the neurons when they are stimulated with extracellular electrodes. However, at present these variables and mechanisms are not accurately known to faithfully reproduce the electrical behavior of the cochlea. In contrast, our work approaches the problem “phenomenologically”, that is, adjusting the model to reproduce the clinical data.

Note that it is not currently possible to reproduce the clinical ECAP with a “deterministic” model due to, among other things, the great variability of clinical ECAP. The novelty of the model is the phenomenological approach of fitting simulated ECAPs by using weights that are obtained from a probability distribution.

Among the most relevant data to fit the electrical parameters of a computational model of a cochlea are the impedance matrix and the registers of the ECAP. The transimpedance matrix allows us to fit the electrical behavior of the model. The ECAP is used to conform the model to reproduce the patient’s neural response.

There are a lot of works dealing with the modelization of CIs. For example, in [6, 18–21, 24] the authors develop a FEM volume conduction model (VC) to predict the electrical fields inside a stimulated cochlea and to analyze the neural response of the ANFs. In [25] it is analyzed the effect of the position of the electrodes and the conductivities of different tissues on the potential distribution. The work of Hanekom [26] presents an extensive review of the 3D modelling of CI, and its applications.

Currently, there are different computational models of CIs that have been fitted using clinical data, such as transimpedances, cochlear dimensions, electrode locations, and loudness and pitch perception [10, 25, 27, 28], and speech understanding [29]. Additionally, there are other models that simulate ECAPs responses [14, 16, 22, 30–32] as the one proposed in this work. In [30], ECAP is modeled using transfer functions that multiply the temporal derivatives of the membrane potential and are calculated using FEM. Moreover, [22] proposes a circuit model whose parameters are calculated by FEM.

The amplitudes of the ECAPs can vary significantly from one patient to another. The main difference between the above mentioned models and ours is the ability of our model to fit the ECAPs of a specific patient. Another fundamental difference is that this fitting is performed using weights that depend on the current densities. This dependency is what gives our model a predictive character. For example, a variation in the design of the CI could lead to a variation in the current densities that reach the neurons and, therefore, a change in the response of the model. The weights are calculated using a computational intelligence optimization method (evolutionary algorithm).

The aim of our computational model is to reproduce the clinical ECAP generated by the auditory nerve fibers (ANFs) when they are stimulated by a CI.

## Method

Our model fits the clinical data of a specific patient. The prototype consists of a set of line sources, the virtual neurons (VNs), propagating the membrane currents given by Hodgkin-Huxley-type (H-H) models, (see e.g., [33–35]). The extracellular potential generated by the VN is calculated by FEM. Each VN contributes with a suitable weight to the simulated potential. The weights are assessed using a cumulative beta distribution characterized by two shape parameters. These parameters are chosen to fit the model to ECAP of the patient. The number of VNs must be large enough to determine, with sufficient precision, the extent of the auditory nerve stimulated around the active electrode. The fitting of the model to the clinical data is done using a differential evolution algorithm. As a result, we obtain a customized model of the neural response produced by the electric stimulation of the CI. The electrodes are stimulated in monopolar mode.

The density of real neurons in the auditory nerve is far greater than the density of VNs in our computational model, therefore, each VN represents a large number of real neurons. The weight of a VN can be understood as the number of real neurons, represented by each VN, that are effectively activated.

In ECAP recording, an active electrode *E*_*s*_ produces a current stimulus that elicits action potentials in the surrounding ANFs. These action potentials are registered by the recording electrode *E*_*r*_. Our model establishes a connection between the current injected by *E*_*s*_ and the potential registered by *E*_*r*_. Our model connects the stimulation current at *E*_*s*_ with the computed ECAP, the potential generated by the VN and registered at *E*_*r*_. The procedure involves two types of FEM computations and a parameter adjustment by using differential evolution (DE):

- Electrode mode: Finite Element computation of current densities at the VNs: A 3D FEM model is constructed to calculate the electrostatic potential and the currents produced by the active electrode *E*_*s*_. This potential determines the current densities at the VNs.
- Neuron mode: Finite Element computation of electric potential at *E*_*r*_: The FEM model is similar to the previous one, but in this case the sources are the VNs. That is, line sources propagating the membrane currents given by Hodgkin–Huxley-type models.

**Fig 1:**
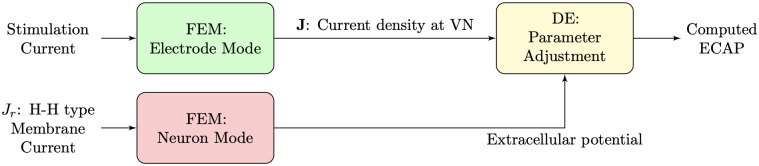
The three main stages of the computational model.

The main stages of the computational model are shown in Fig 1.

### Ethics statement

The ethics committee of the Complejo Hospitalario Universitario Insular Materno Infantil approved the study prior to the data collection process. All patients agrees to participate in the study by signing the informed consent.

### FEM model

#### Domain description

Our study is focused on the small region around the stimulating electrode. For that reason, it is not essential to construct a complete spiral-shaped representation of the cochlea. Nevertheless, as each tissue has a different conductivity, it is important that the model represents correctly the different structures that conform the cochlea.

The cochlear model is delimited by a 20 mm radius sphere containing a medium whose conductivity, *σ*_*ext*_, is chosen to fit the values of the transimpedances of the model to the ones provided by clinical data, thus guaranteeing a reasonable electrical behavior of our simplified model.

The transimpedance matrix is obtained by measuring the potential profile recorded along all contacts when a single contact is stimulated in monopolar mode. The element *Z*_*ij*_ of the transimpedance matrix is given by the ratio *V*_*i*_/*I*_*j*_, where *V*_*i*_ is the voltage at the electrode *i* respect to the reference electrode (ground) and *I*_*j*_ is the current delivered by the stimulating electrode *j*. The transimpedance matrix was recorded from a specific patient using the following configuration of the Custom Sound Evoke Potentials software from Cochlear (Cochlear Ltd. Sydney, Australia). A biphasic stimulus with a phase width of 25 μs, an interphase gap width of 8 μs and a frame period of 122 μs was set. The recording time was set at the end of the first phase. The stimulation current level was 0.636 mA. The ball electrode (MP1) was used as reference in the stimulation circuit and the implant case (MP2) as reference in the recording circuit. The neural responses were carried out with Custom Sound Evoke Potential Software tool (version 5.2).

The simulated impedances depend on the conductivity of the medium, so a change in *σ*_*ext*_ causes a variation in the computed values of *Z*_*ij*_. Our objective is to determine the value of *σ*_*ext*_ that adjusts the simulated impedances to the real ones.

Our computational model represents a section of the cochlea with a total length of 9 mm. In this case, we focus on the basal portion of the cochlea selecting the first six values *Z*_*patient*_ = {*Z*_11_, *Z*_21_, …, *Z*_61_} of transimpedance matrix, where the active electrode, *j* = 1, is the most basal electrode. The values of these impedances are *Z*_*patient*_ = {2922.99, 870.15, 785.55, 700.95, 647.99, 616.35} Ω. We could also have chosen any other active electrode for our adjustment with a similar result. The simulated values of the these impedances, with *σ*_*ext*_ = 0.3 S m^−1^, are *Z*_*simulated*_ = {1913.7, 851.41, 736.15, 696.59, 675.74, 662.37} Ω, taking the stimulating electrode, *E*_*s*_, as the central one.

The reference electrode is placed close to the boundary of the delimiting sphere and its radius is *r* = 0.5 mm.

**Fig 2:**
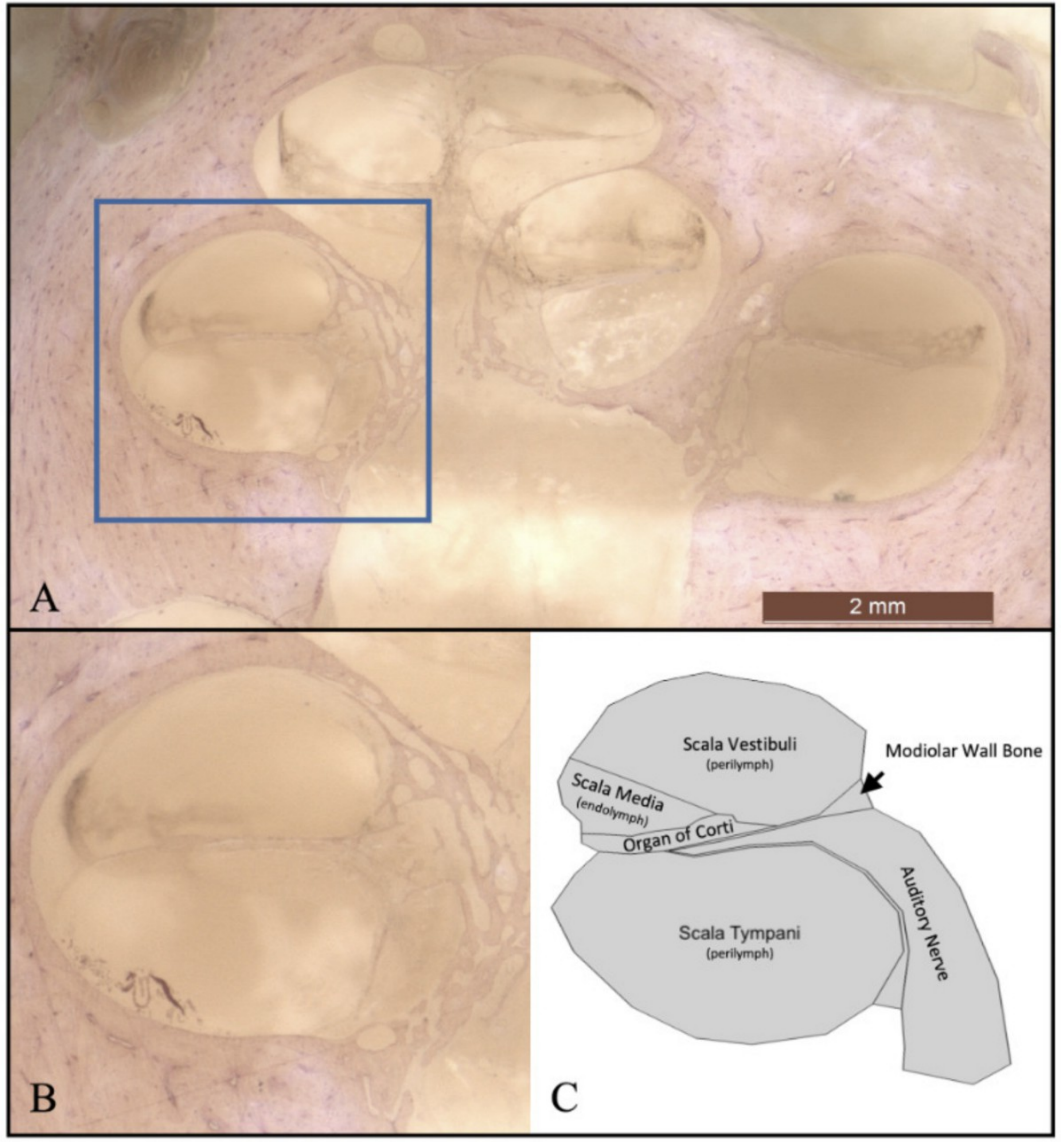
A section of the cochlea. This figure displays an histological section of the cochlea for light microscope of a fresh temporal bone using the paraffin technique. The histological section was performed parallel to the modiolar axis. Full view of the histology section of the cochlea (A). Zoom view of the region of interest of the cochlea (B). Geometry generated based on histology image (C).

The computational model of the cochlea has been generated by histological data from temporal bone shown in Fig 2. The histological technique used is described in reference [36].

**Fig 3:**
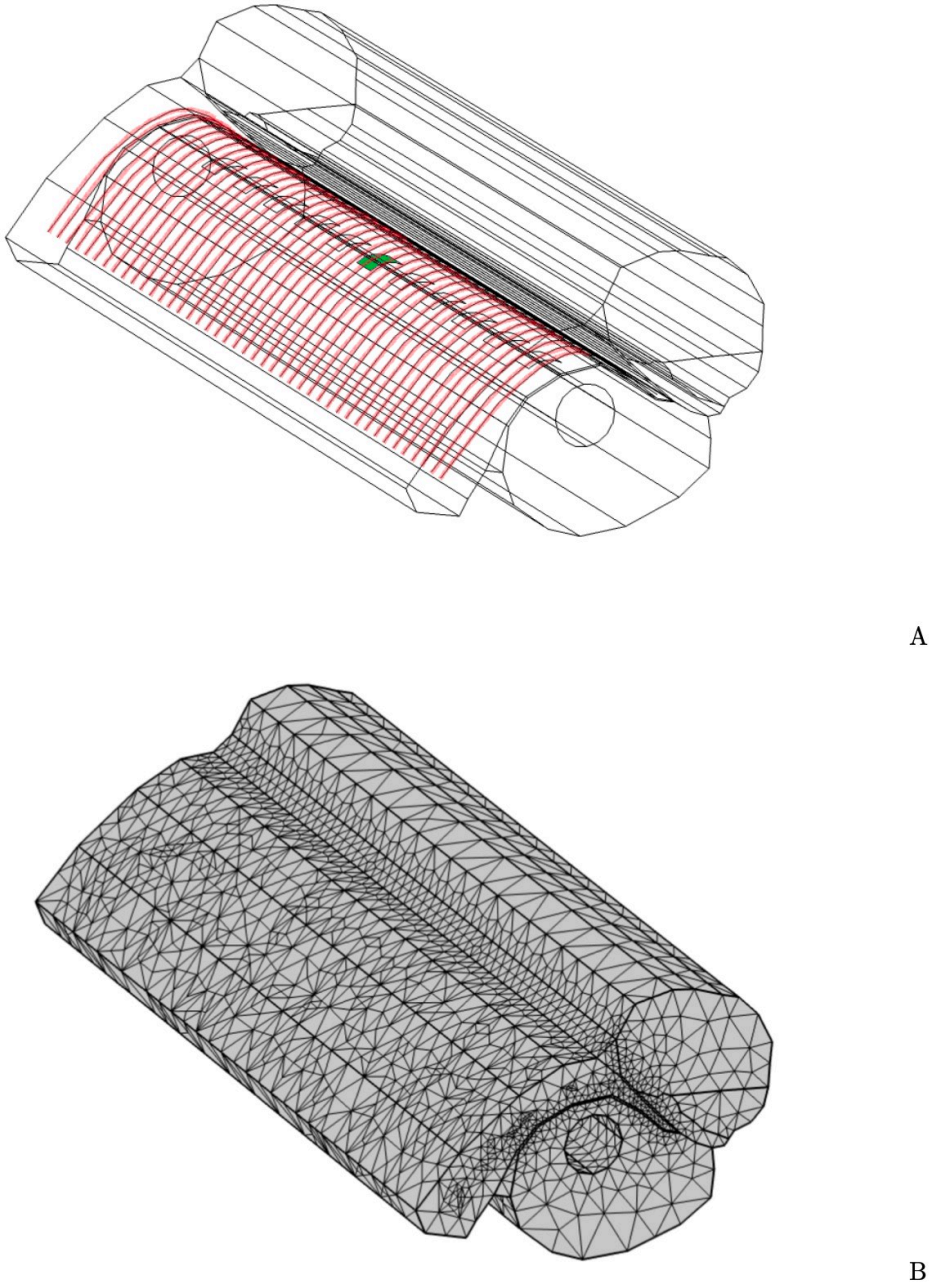
Electrode array and virtual neurons. Straight section of the cochlea containing the half banded electrode array and the virtual neurons. The stimulating electrode is shown in green and the virtual neurons are shown in red (A). Surface mesh with a refinement around the VN (B).

Fig 3A shows the straight section of the cochlea, the VNs and the electrode array used in this work. The mesh of this geometry is shown in Fig 3B. The sphere delimiting the domain is not represented in this figure.

The computational model contains 11 electrodes. They are embedded in a silicone carrier, that is a good electrical insulator, so the current inside the carrier is negligible and it can be removed from the domain. In this case the surface of the silicone constitutes the internal boundary of the domain and it is considered as an isolating boundary. Thus, the boundary of the domain Ω is formed by an exterior boundary, the sphere, *∂*Ω_*ext*_, and the interior boundary, *∂*Ω_*int*_, that is, *∂*Ω = *∂*Ω_*ext*_ ∪ *∂*Ω_*int*_.

The conductivities of the different tissues considered in this work are: *σ*_endolymph_ = 1.67 S m^−1^, *σ*_perilymph_ = 1.42 S m^−1^, *σ*_auditorynerve_ = 0.3 S m^−1^, *σ*_organ of corti_ = 0.012 S m^−1^, *σ*_modiolar wall bone_ = 0.2 S m^−1^ and *σ*_ext_ = 0.3 S m^−1^. A review of the resistivities given by some authors is shown in appendix E of [37]. Additionally, a detailed study of impedances of the cochlear structures can be found in [38].

The number of VNs of our model is *N* = 43 and they are separated by 0.21 mm from each other.

The radius of the electrode array is 0.3 mm, the dimensions of the electrodes are 0.7 mm × 0.3 mm and the inter-electrode distance is 0.7 mm. This design is based on the CI512 electrode array from Cochlear (Cochlear Ltd. Sydney, Australia). We have used Comsol Multiphysics 5.6 with quadratic tetrahedral elements for FEM calculations. The number of tetrahedral elements of the complete domain is 866 309, the average quality (mean ratio) is 0.735 and the minimum element quality is 0.043. The number of degrees of freedom is 1171 692.

It is important to highlight that the size of the elements at the VNs must be small enough to accurately reproduce the potential abruptly fluctuating in time and space. The mesh around the VNs has been refined 3 times, giving rise to a mesh with edges of 0.018 mm at the VNs.

#### Formulation of the problem

Although we are dealing with a dynamic problem, the involved frequencies are very low. The stimulus rate of CI is around 1 kHz and the conduction velocity of the ANF is around 15 m/s [39, 40]. Therefore, the problem can be solved by using a steady current approach [41]. The potential *ϕ* is given by

$$\begin{array}{c} \nabla \cdot \sigma \nabla \phi =-S,\;\text{in}\;\Omega \end{array}$$ (1)

where *σ* is the conductivity and *S* is the volume current source (see for example [42]). This approach, widely used, is known as the volume conduction model. Here, Ω is the domain formed by the cochlea and the surrounding sphere and it is constituted by different structures characterized by their conductivities *σ*_*i*_, that we assume to be constant. The current density in each structure is given by **J** = *σ*_*i*_**E**, with **E** = −∇*ϕ*.

The source depends on whether the FEM is actuating as electrode or neuron mode.

Electrode mode:

The active electrode *E*_*s*_ is a surface delivering a net current *I*_0_. As *E*_*s*_ is part of the inner boundary, it is modeled as a Neumann boundary condition and the source *S* = 0 is null. The other electrodes are considered as floating potentials.

Although in an ECAP recording the input current takes several values, it is not necessary to solve a FEM problem for each current. The linearity of the problem allows us to accomplish a unique FEM simulation for a given *I*_0_. Thus, if *I*_*k*_ = *c*_*k*_*I*_0_ is the input current at *E*_*s*_ and *c*_*k*_ is the proportionality constant between both currents, the solution of Eq (1) for *I*_*k*_ is *ϕ*(*I*_*k*_) = *c*_*k*_*ϕ*(*I*_0_), being *ϕ*(*I*_0_) the solutions of (1) for *I*_0_. The currents considered here are the amplitudes of the stimulus pulse of the CI, so the problem is strictly steady.

Neuron mode:

In this case, we can consider each VN as a line current source with a spatio-temporal dependence given by λ(*t*, *s*) = λ(*t* − *v*^−1^ *s*), where *s* is the arc length of VN and *v* is the conduction velocity in the ANF. The line current density λ(*t*) is inferred from the Hodgking-Huxley model for an ANF from [35]. The determination of λ(*t* − *v*^−1^ *s*) will be detailed in a subsection below. The line current source is given by

$$\begin{array}{c} S\left(x,y,z,t\right)=\int_{C}\lambda \left(t,s\right)\delta \left(x-X\left(s\right)\right)\delta \left(y-Y\left(s\right)\right)\delta \left(z-Z\left(s\right)\right)ds \end{array}$$ (2)

where *C* is the parametric curve (*X*(*s*), *Y*(*s*), *Z*(*s*)) describing the VN and *s* the arc length. Each VN is represented by a source *S*_*n*_(*x*, *y*, *z*, *t*) with a line current λ_*n*_(*t*, *s*).

### Boundary conditions

#### Active electrode

The active electrode *E*_*s*_ is implemented as the Neumann boundary condition

$$\begin{array}{c} J\cdot n=-\sigma \frac{\partial \phi }{\partial n}=\frac{I_{0}}{A}\;\text{on}\;E_{s}, \end{array}$$ (3)

where **J** is the current density, **n** is the unitary normal vector to *E*_*s*_, *I*_0_ is the current delivered by the active electrode, *A* its surface, and *σ* the conductivity of the surrounding medium.

#### Disconnected electrodes

The electrodes not actuating as terminals are disconnected. They must be considered as floating potentials because they are equipotential surfaces with an unknown potential. This type of boundary condition can be implemented using the linearity of the problem. As example, let us consider one active electrode, *E*_1_, injecting a current *I*_0_, and other electrode, *E*_2_, disconnected (*I* = 0). Now, let *ϕ*_1_ be the solution of the potential problem (1) when the electrode *E*_1_ is at 1 V and electrode *E*_2_ is at zero potential. Reciprocally, let *ϕ*_2_ be the solution of (1) when the electrode *E*_1_ is at zero potential and electrode *E*_2_ is at 1 V. Taking into account the linearity of the problem we can write the general solution of (1) as:

$$\begin{array}{c} \phi =\alpha_{1}\phi_{1}+\alpha_{2}\phi_{2}. \end{array}$$ (4)

The coefficients *α*_1_ and *α*_2_ can be calculated imposing the restrictions of our particular problem. Thus, integrating Eq (3) on the electrodes *E*_1_ (active) and *E*_2_ (floating), and imposing the conditions $-\sigma \oint_{E_{1}}n\cdot \nabla \phi \;da=I_{0}$ and $-\sigma \oint_{E_{2}}n\cdot \nabla \phi \;da=0$, we obtain:

$$\begin{array}{c} \alpha_{1}I_{11}+\alpha_{2}I_{12}=I_{0} \end{array}$$ (5)

$$\begin{array}{c} \alpha_{1}I_{21}+\alpha_{2}I_{22}=0, \end{array}$$ (6)

being $I_{nm}=-\sigma \oint_{E_{n}}n\cdot \nabla \phi_{m}\;da$. The solution is given by (4) after solving the unknown coefficients *α*_1_ and *α*_2_ of the above equation system.

### Boundary condition at the surrounding sphere and reference electrode

#### Electrode mode

In this case the currents flow from the active electrode, *E*_*s*_, to the reference one, taken as ground (*ϕ* = 0). The surrounding sphere is considered to be an isolating surface (**J** ⋅ **n** = 0), so that the boundary condition is

$$\begin{array}{c} \frac{\partial \phi }{\partial n}=0\;\text{on}\;\partial \Omega_{ext} \end{array}$$ (7)

#### Neuron mode

Now, the neurons act as potential sources and the small currents emanating from them are diffused in the medium. In this situation we consider the reference electrode as a floating potential and the surrounding sphere is modeled as an asymptotic boundary condition that mimics an unbounded domain with null potential at infinity (see e.g., [43, 44]). We have imposed a first order asymptotic boundary condition (ABC) on the sphere *∂*Ω_*ext*_.

$$\begin{array}{c} \frac{\partial \phi }{\partial n}+\frac{\phi }{R}=0,\;\text{on}\;\partial \Omega_{ext} \end{array}$$ (8)

This is a homogeneous Robin boundary condition, where *R* = 20 mm is the radius of the sphere limiting the domain. The error introduced by this approximation is *O*(*R*^−3^) and it is accurate enough for our purpose.

### Isolating surfaces

The boundary of the silicone carrier is taken as an isolating surface, and therefore, in the part of *∂*Ω_*int*_ not occupied by the electrodes, we have

$$\begin{array}{c} \frac{\partial \phi }{\partial n}=0 \end{array}$$ (9)

### Modeling of the neural response

#### Line source approximation for computing the extracellular potential

The line source approximation (LSA) is an easy and accurate approach to compute the extracellular potential at any point in space using the values of the membrane currents [45–49]. Usually, LSA is used to calculate an analytic expression of the electric potential produced by line segments in an homogeneous medium. In this work it is applied to deal with lines having a continuous spatio-temporal variation in a non homogeneous medium.

In a myelinated nerve fiber the highest current concentration takes place in the nodes of Ranvier (see, for example, chapter 6 of [50]). Thus, the modelization of a unique nerve fiber could be performed by placing punctual current sources in the positions of the nodes of Ranvier. Nevertheless, each VN of our model represents a great number of myelinated nerve fibers (about 100) whose nodes of Ranvier can be considered as randomly distributed along the VN. For this reason, an approach closer to reality is to take the VN as a line source current with a continuous linear density. Furthermore, this approach presents few difficulties when generating the FEM mesh.

**Fig 4:**
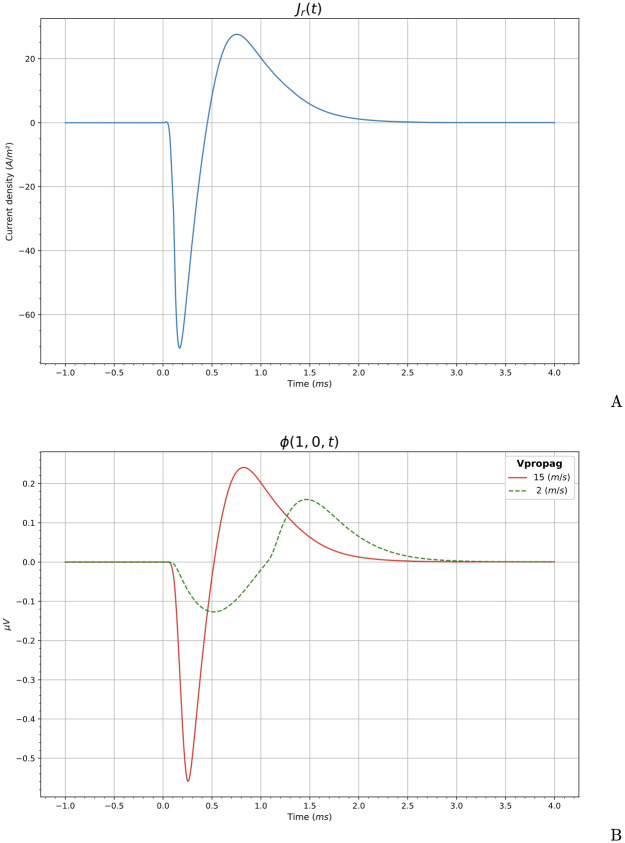
Membrane current density and extracellular potential. This figure shows the membrane current generated in an ANF, used as input in our model (A). Extracellular potentials calculated for an academic example at *r* = 1 mm and *z* = 0 mm corresponding to velocities of propagation of 2 m s^−1^ (dashed green) and 15 m s^−1^ (red) are shown in (B).

The current density normal to the surface at the nodes of Ranvier for an ANF, *J*_*r*_(*t*), is the membrane current (ionic and leak) density, obtained from the type H-H (Wang-Buzsaki) model [51] implemented in [35] (see Fig 4A).

This current density allows us to calculate an equivalent linear current density λ(*t*) of a VN. Let us consider an ANF with the following morphology: the total length is *l* = 2.7 mm and it has *N*_*r*_ = 135 nodes of Ranvier with a diameter *d* = 2 μm and length *h* = 1 μm (see [40] for a discussion about the ANF morphology). The internodes length is 200 μm. The extracellular current source is located at 1 mm from Ranvier node 20 with a negative stimulating current of 2 mA during 100 μs. The membrane current density, *J*_*r*_(*t*), is calculated at node 22. Then, the total charge passing across the membrane in the nodes of Ranvier per unit of time is *J*_*r*_(*t*)*N*_*r*_*πdh*, and the equivalent linear current density is λ(*t*) = *J*_*r*_(*t*)*N*_*r*_*πdh*/*l* = 4.65 × 10^−8^ *J*_*r*_(*t*) A m^−1^.

The propagation throughout the neuron is explicitly introduced by using the expression λ(*t* − *v*^−1^ *s*), where *v* is the propagation velocity and *s* is the arc length of the trajectory of the neuron. A similar approach is used in [52], but considering discrete sources, placed at the Ranvier nodes, instead of a current line.

In order to show the influence of the propagation velocity in the shape of the extracellular potential, let us consider the following academic example consisting in a line extended from (0, 0, −1) mm to (0, 0, 1) mm. In this case, the arc length is *s* = 1 + *ζ*. The explicit calculation of the potential at any point in space due to the linear current density λ(*t* − *v*^−1^ *s*) = 6.27 × 10^−8^ *J*_*r*_(*t* − *v*^−1^ *s*) A m^−1^ gives

$$\begin{array}{c} \phi \left(r,z,t\right)=\frac{1}{4\pi \sigma }\int_{-1}^{1}\frac{\lambda (t-v^{-1}\left(\zeta +1\right))}{\sqrt{{\left(\zeta -z\right)}^{2}+r^{2}}}d\zeta , \end{array}$$ (10)

where *J*_*r*_(*t*) is the membrane current density (see Fig 4A), *r* and *z* are cylindrical coordinates of the the field point, *ζ* is the source point along the line, and *σ* = 1 S m^−1^ is the conductivity of the medium. The resulting potentials at cylindrical coordinates *r* = 1 mm and *z* = 0 mm, for velocities *v* = 2 m s^−1^, and *v* = 15 m s^−1^, are shown in Fig 4B. Note that an increase in the propagation velocity implies a greater amplitude of the ECAP. In addition, the increase in the velocity causes the duration of the ECAP to decrease, being more similar to that of the stimulus.

In our FEM model we have implemented *N* = 43 VNs (see Fig 3A). The linear current density of the *n*th VN, described by the curve *C*_*n*_, is:

$$\begin{array}{c} \lambda_{n}\left(t-v^{-1}s\right)=w_{n}\left(N_{r}\pi dh/l\right)J_{r}\left(t-v^{-1}s\right), \end{array}$$ (11)

where *w*_*n*_ is the weight assigned to this VN. We have taken a propagation velocity *v* = 15 m s^−1^ in the ANF [39, 40]. This velocity value is also in consonance with the results obtained in [53]. In general, it is not possible to get an analytic expression for the arc length *s* of *C*_*n*_. To overcome this difficulty, we have constructed a parameterization *s*(*x*) by interpolating the numerical computation of true length for a set of *x* coordinates.

**Fig 5:**
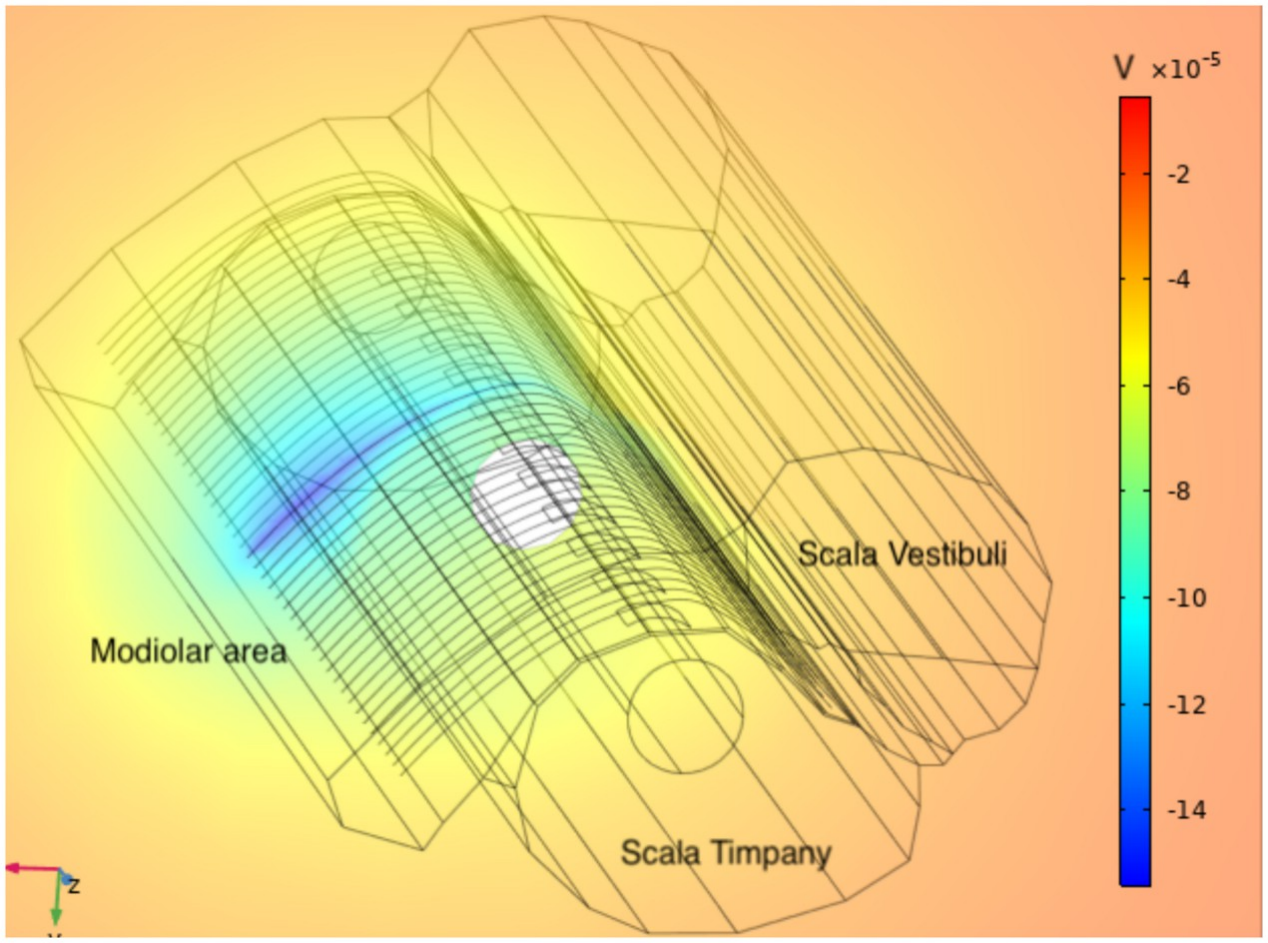
Potential. Representation of the potential generated by the VN placed in the central position at *t* = 0.4 ms.

In Fig 5 it is shown the potential generated at t = 0.4 ms when all the VN are turned off except the central one.

#### Determination of the weights

Our model must reproduce the clinical data as accurately as possible. The only measure of neuronal activity that can be recorded with a CI is the action potential registered at the recording electrode *E*_*r*_, that is, the ECAP. The objective of the ECAPs is double. Firstly, we want to know if there is a neural response of the region of the auditory nerve close to the stimulating electrode. The second aim is to detect the current intensity threshold that evokes an action potential. For this last reason, the ECAPs are carried out for several current intensities, *I*_*k*_. We have to determine the weight *w*_*n*_ that must be assigned to each VN so that the amplitude of the potential provided by the model coincides with the amplitude of the action potential registered by the ECAP. The amplitudes are the difference between negative N1, and positive P2 peaks for both real and simulated action potentials.

**Fig 6:**
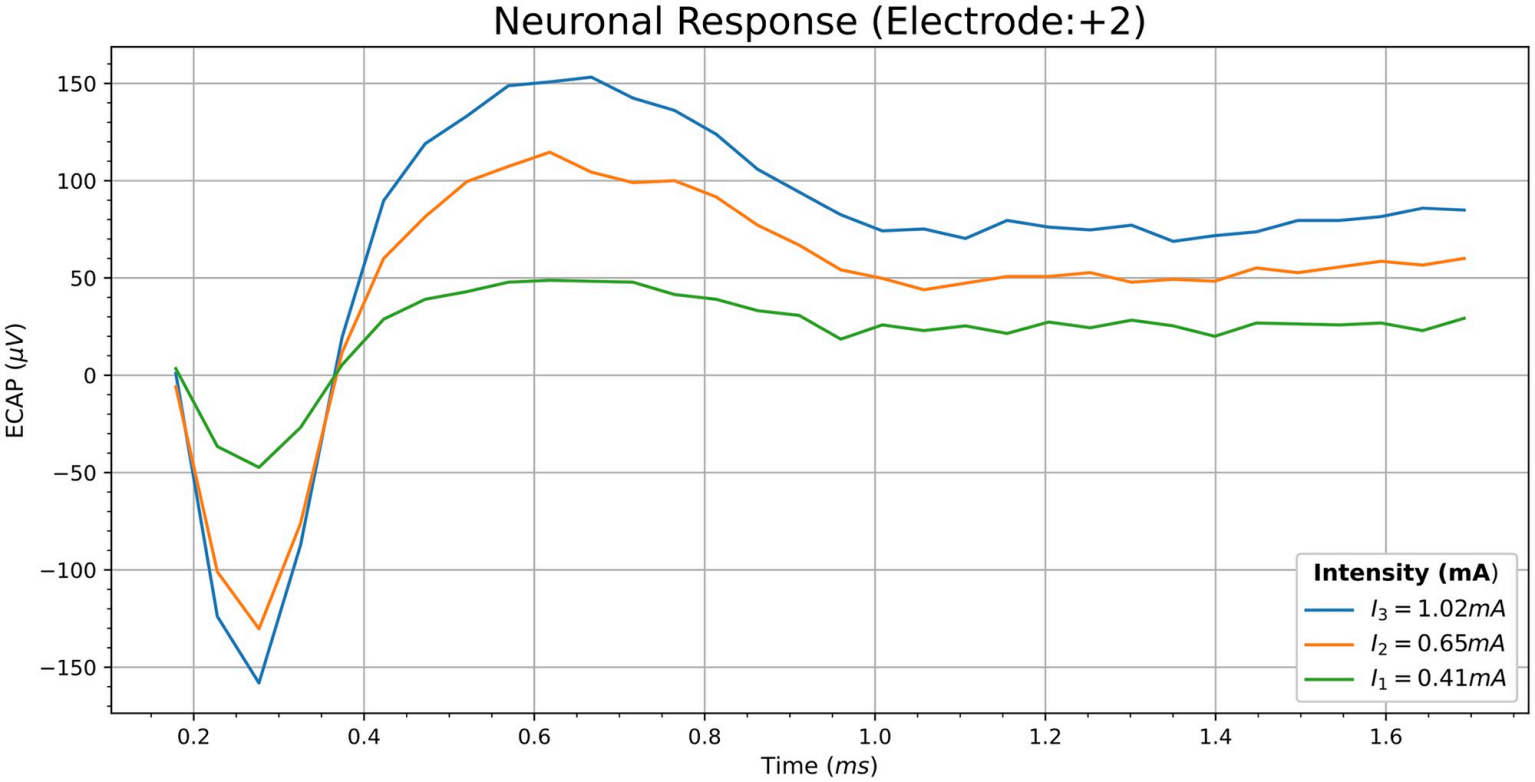
Real ECAPs produced by three stimulation currents. This figure shows the resulting ECAPs, provided by Custom Sound Evoke Potentials software from Cochlear, after the following stimulation currents: *I*_1_ = 0.41 mA (green line), *I*_2_ = 0.65 mA (orange line) and *I*_3_ = 1.02 mA (blue line).

First of all, a remark about the enumeration of the electrodes should be pointed out. As our computational model only represents a portion of the real CI, we always take the stimulating electrode *E*_*s*_ as the central one and it will be considered as electrode 0. The rest of electrodes are enumerated with positive or negative numbers, depending on the relative position with respect to the central one. The local numbering adopted for the real CI is similar to the numbering of the model (considering *E*_*s*_ as electrode 0). The numeration of the VNs follows an analogous criterion: the VN over the stimulating electrode is taken as 0 and the rest of VNs are enumerated with positive or negative numbers. Fig 6 shows three real ECAPs corresponding to three stimulation currents delivered by a basal electrode. In this case, the recording electrode is placed two electrodes away from the stimulating one (electrode 2 in our local numbering).

**Fig 7:**
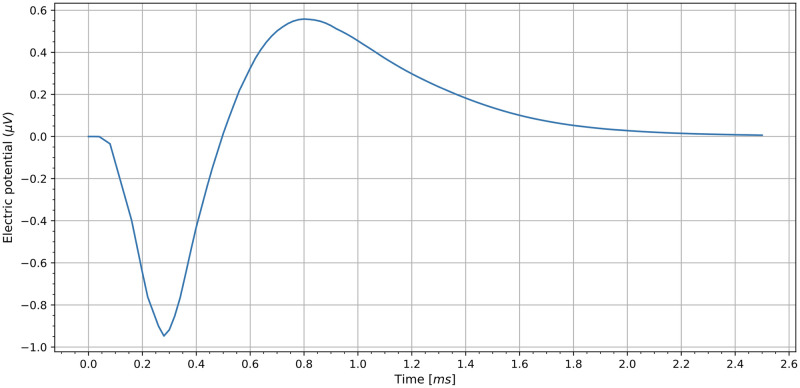
ECAP generated by VN number 0 at electrode 2 for a propagation velocity *v* = 15 m s^−1^.

We denominate $\phi_{ECAP}^{e}\left(I_{k}\right)$ to the amplitude of the action potential registered by the ECAP at the *e*th electrode when the stimulating electrode *E*_*s*_ supplies a current *I*_*k*_. $\phi_{n}^{e}$ is the amplitude of the simulated potential produced by the *n*th VN and measured at the electrode *e*th, when the *n*th VN is turned on and the other VNs are turned off. We assume in this situation that all the active VNs are fed with the same linear current density. As an example, in Fig 7 it is shown the potential computed at electrode 2 (two electrodes away from *E*_*s*_) when all the VNs, except the central one, are turned off. Comparing Figs 6 and 7 it can be seen that the shapes of both ECAPs are similar. Furthermore, the time lapse between N1 and P2 is also similar, being around 0.4 ms for real ECAPs and 0.5 ms for the computed ECAP.

The weight $w_{n}^{e}\left(I_{k}\right)$ by which $\phi_{n}^{e}$ must be multiplied to be equal to $\phi_{ECAP}^{e}\left(I_{k}\right)$ is given by:

$$\begin{array}{c} \phi_{ECAP}^{e}\left(I_{k}\right)=w_{n}^{e}\left(I_{k}\right)\phi_{n}^{e}. \end{array}$$ (12)

This equation reflects that we only need one VN to reproduce the amplitude of the ECAP measured at a given electrode *e*. However, a computational model with a unique VN is not able to specify what region of the auditory nerve is affected after the stimulation of the active electrode. To be able to discriminate which part of the auditory nerve is activated, we need a considerable number of VNs. If we have *N* VNs and a unique ECAP measure, there are *N* − 1 extra degrees of freedom (weights) that must be determined. This is the usual situation, in which the ECAP measure is carried out at the electrode +2 (two electrodes away from the stimulating one). If we multiply Eq (12) by an arbitrary parameter *δ*_*n*_ and sum for all the VNs we obtain:

$$\begin{array}{c} \sum_{n=-N^{\prime }}^{N^{\prime }}\delta_{n}\phi_{ECAP}^{e}\left(I_{k}\right)=\sum_{n=-N^{\prime }}^{N^{\prime }}\delta_{n}w_{n}^{e}\left(I_{k}\right)\phi_{n}^{e}, \end{array}$$ (13)

where $N^{\prime }=\frac{N-1}{2}$, being *N* the number (odd) of VNs. Writing $\Delta =\sum_{n=-N^{\prime }}^{N^{\prime }}\delta_{n}$ we deduce from the equation above:

$$\begin{array}{c} \phi_{ECAP}^{e}\left(I_{k}\right)=\sum_{n=-N^{\prime }}^{N^{\prime }}\frac{\delta_{n}}{\Delta }w_{n}^{e}\left(I_{k}\right)\phi_{n}^{e}=\sum_{n=-N^{\prime }}^{N^{\prime }}{\bar{w}}_{n}^{e}\left(I_{k}\right)\phi_{n}^{e}, \end{array}$$ (14)

where ${\bar{w}}_{n}^{e}=\frac{\delta_{n}}{\Delta }w_{n}^{e}$ are the new weights. Eq (14) shows that the potential registered at electrode *e* is the sum of the weighted contributions of the potentials produced for each VN. The arbitrary parameters *δ*_*n*_ represent the extra degrees of freedom, introduced by the additional VNs, that will be related with a proper physical magnitude.

Suppose that for a selected number of patients we have a number of ECAPs samples *N*_*d*_ > 1 for each current *I*_*k*_. We intend to train our model with these samples in order to be valid in the usual situations, where we only have a unique ECAP. Our objective is twofold. Using the expression (14), we want to interpolate the amplitude of the ECAP for a given electrode, named *anchor electrode*. This electrode is the one for which the computed and measured amplitudes of the ECAPs are enforced to be identical. In addition, we want the difference between the ECAP amplitudes provided by the clinical data and those calculated by the model to be as small as possible in the remaining electrodes. The achievement of the latter objective lies in the proper choice of the *δ*_*n*_ coefficients.

Specifically, let *a* be the anchor electrode. Following the Eq (12) we calculate the weights $w_{n}^{a}\left(I_{k}\right)$ using the data $\phi_{ECAP}^{a}\left(I_{k}\right)$. Introducing these weights in the second term of Eq (14) we have:

$$\begin{array}{c} \phi_{ECAP,comp}^{e}\left(I_{k}\right)=\sum_{n=-N^{\prime }}^{N^{\prime }}{\bar{w}}_{n}^{a}\left(I_{k}\right)\phi_{n}^{e}, \end{array}$$ (15)

where ${\bar{w}}_{n}^{a}=\frac{\delta_{n}}{\Delta }w_{n}^{a}$. This is the expected amplitude of the potential at *e*th electrode, but computed from the ECAP given at the anchor electrode *a*. Obviously, if *e* = *a* we have $\phi_{ECAP,comp}^{e}\left(I_{k}\right)=\phi_{ECAP}^{e}\left(I_{k}\right)$, but this is not true for the rest of electrodes. The objective is to find the parameters *δ*_*n*_ that minimize the difference between $\phi_{ECAP,comp}^{e}\left(I_{k}\right)$ and $\phi_{ECAP}^{e}\left(I_{k}\right)$ using the remaining *N*_*d*_ − 1 data. In the next subsection we will explain how to calculate these parameters.

#### Relation of *δ*_*n*_ with the current density

The usefulness of a computational model is linked to its ability to predict what happens when electrical or structural changes occur in the CI, such as the type of electrode stimulation (bipolar, tripolar, etc.), the geometric design of the electrodes [12], the distance between the electrode array and the auditory nerve (perimodiolar vs lateral implant), etc. The only way for the model to be sensitive to these changes is to link the weights to some physical variable that varies when the design changes. For the reasons outlined below, we consider this variable to be the current density norm.

**Fig 8:**
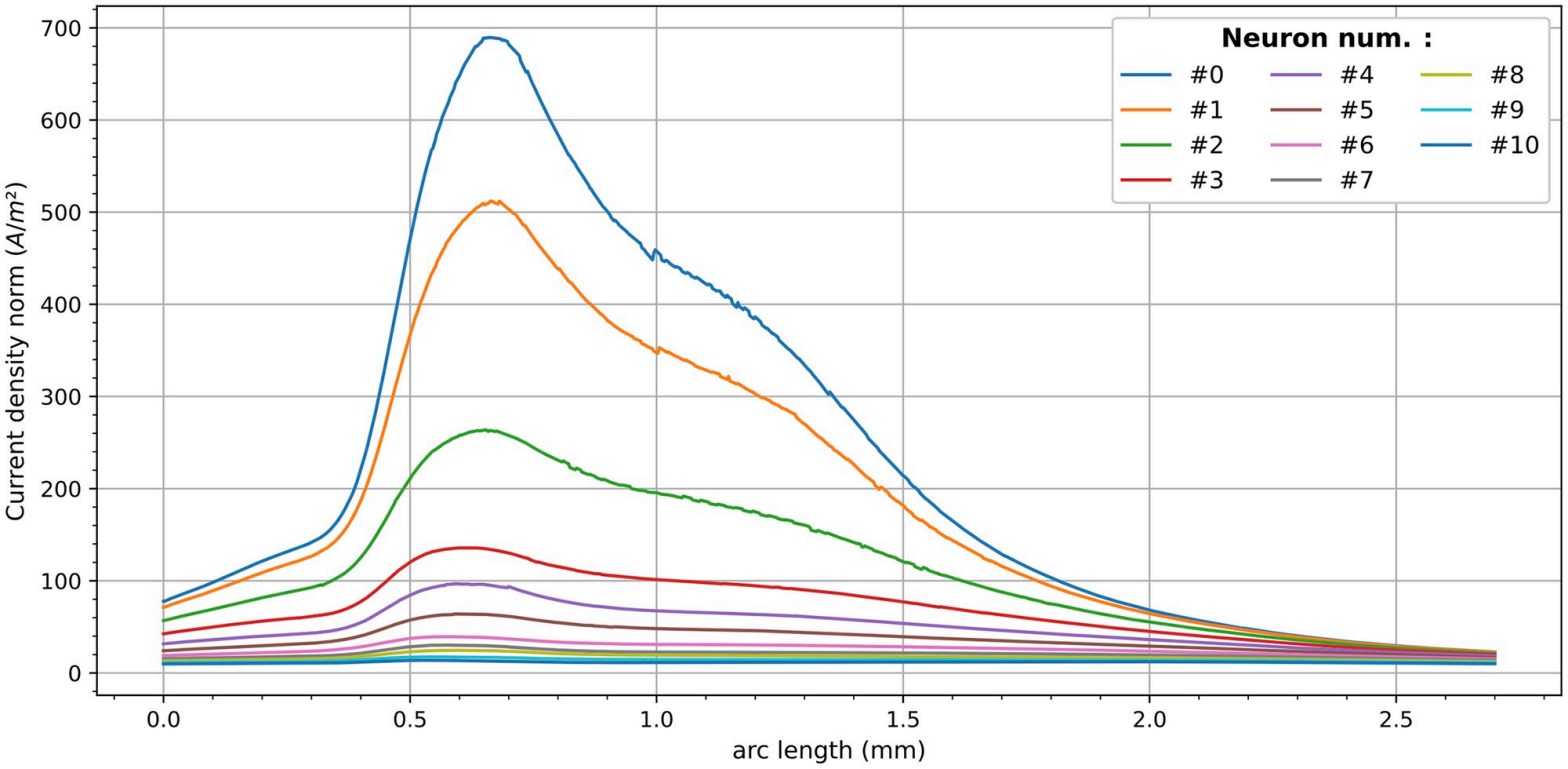
Current densities at the first 11 VN when *E*_*s*_ supply a current of −1 mA.

The input current establishes a distribution of potential and current density along the VNs. In Fig 8 it is shown the current densities at the first eleven VNs (from 0 to 10) when the stimulating electrode supplies a current of −1 mA. Most of the models for neural simulation attribute the activation of the neuron to the difference of potentials along the axon [18, 54]. Nevertheless, the most accurate electrodiffusion models based on the Poisson-Nernst-Planck attribute the trigger of an action potential to the ion currents normal to the surface of the fibers [47, 50]. An experimental study of how the orientation of the electric field affects the central nervous system neurons is presented in [55]. In myelinated fibers the ionic flux takes place in the nodes of Ranvier. It is extremely difficult to know with precision which is the normal vector to the Ranvier nodes in the cochlear nerve. As our VNs represent a set of real ANF, we consider more realistic to link the probability of excitation of a nerve fiber to the norm of the current density, *J* = |**J**|, generated by *E*_*s*_. However, *J* variates along the VN, but we need to assign a unique weight to each VN.

It seems reasonable to think that the higher the current density reaching the VN, the greater should be *δ*_*n*_. This motivates us to define the parameters *δ*_*n*_ as the probability *P*_*n*_ that an ANF, represented by a VN, is excited. This probability *P*_*n*_ must increase when the current density on the VN increases. This idea is inspired by the paper [15], where the authors use an integrated-gaussian distribution to calculate the probability of discharge of an ANF in terms of stimulus intensity. To calculate, *P*_*n*_, firstly we define the probability per unit length, *p*_*n*_, of a section ANF to be excited with a current density less than or equal to *J*:

$$\begin{array}{c} p_{n}=\frac{F(J;\alpha ,\beta ,J_{max},J_{min})}{L}, \end{array}$$ (16)

where *L* is the length of the VN and

$$\begin{array}{c} F\left(J;\alpha ,\beta ,J_{max},J_{min}\right)=\int_{Jmin}^{J}f\left(X;\alpha ,\beta ,J_{min},J_{max}\right)dX \end{array}$$ (17)

is the cumulative distribution function of a beta distribution probability defined in the interval [*J*_*min*_, *J*_*max*_] (see e.g., [56]) given by:

$$\begin{array}{c} f\left(X;\alpha ,\beta ,J_{min},J_{max}\right)=\frac{1}{J_{max}-J_{min}}\frac{\Gamma (\alpha +\beta )}{\Gamma (\alpha )\Gamma (\beta )}(\frac{X-J_{min}}{J_{max}-J_{min}}{)}^{\alpha -1}(\frac{J_{max}-X}{J_{max}-J_{min}}{)}^{\beta -1}, \end{array}$$ (18)

where Γ is the gamma function, *J*_*min*_ is the current density threshold below which there is no response to the input stimulus, and *J*_*max*_ a maximum current density that will be adjusted to the particular problem. Thus, the probability of excitation of the complete ANF, and our definition of *δ*_*n*_, is:

$$\begin{array}{c} \delta_{n}=P_{n}=\int_{C_{n}}p_{n}ds=\frac{1}{L}\int_{C_{n}}F\left(J;\alpha ,\beta ,J_{max},J_{min}\right)ds, \end{array}$$ (19)

where *C*_*n*_ is the curve described by the *n*th VN and *s* is the arc length along this curve.

Note that, since our model is symmetric with respect to the stimulating electrode, so are the current densities. As a consequence of this symmetry, the delta parameters verify that *δ*_*n*_ = *δ*_−*n*_. However, in general, this is not true for weights ${\bar{w}}_{n}^{a}$.

The choice of the beta distribution is due to the fact that it constitutes a family of continuous probability distributions supported on a finite interval. In practice, we take *J*_*min*_ = 0 and *α*, *β* and *J*_*max*_ are calculated to fit the clinical data using a differential evolution algorithm. The flexibility of the beta distribution makes it very suitable for this purpose.

Once the connection between *δ*_*n*_ and *J* is established, we could interpret the weights ${\bar{w}}_{n}^{a}$ as the number of real neurons of each VN that have been activated.

#### Outline of the model

**Fig 9:**
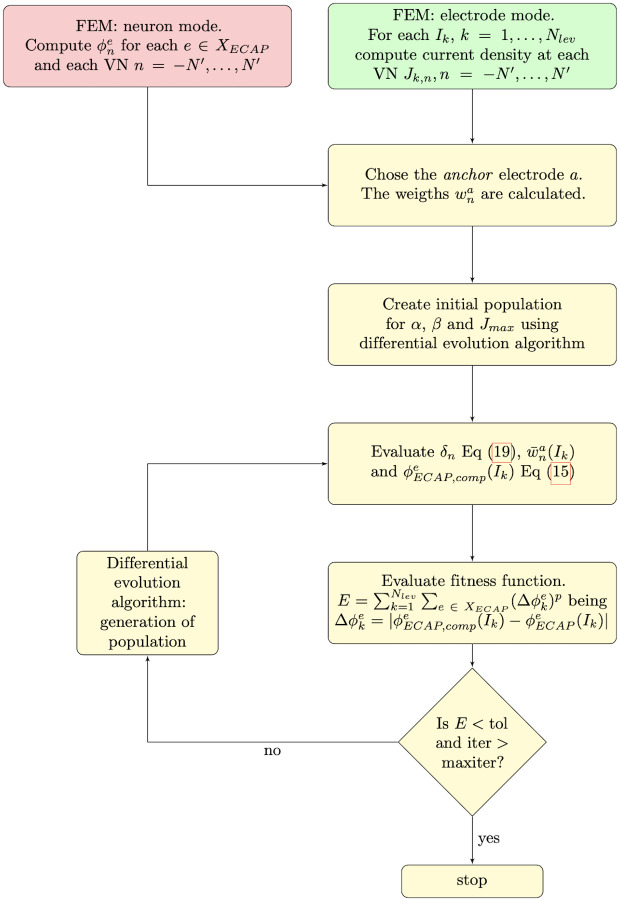
Flowchart of the program.

Let $X_{ECAP}=\left\{e_{1},e_{2},\ldots ,e_{N_{d}}\right\}$ be the set of electrodes where we have an ECAP measure. The flowchart of the program used to fit the model is shown in Fig 9.

1. FEM (electrode mode) is used to calculate the electric potential originated by the stimulating electrode *E*_*s*_ when it supplies the intensities *I*_*k*_, *k* = 1, …, *N*_*lev*_. With these results, the current density at each VN, *J*_*k*,*n*_, *n* = 1, …, *N* is calculated.
2. The electric potential *ϕ*_*n*_(*x*, *y*, *z*) produced by each VN acting alone is evaluated with FEM (neuron mode). Afterwards, the amplitudes $\phi_{n}^{e}$ of the potentials at each electrode where we have ECAP data *e* = 1, …, *N*_*d*_ are computed.
3. A particular *anchor* electrode *a* to interpolate the amplitude is chosen. Additionally, the weights $w_{n}^{a}$ are obtained.
4. The differential evolution algorithm selects new values of *α*, *β* and *J*_*max*_.
5. The parameters *δ*_*n*_ are evaluated using Eq (19). Then, the weights ${\bar{w}}_{n}^{a}\left(I_{k}\right)$ and the computed ECAP $\phi_{ECAP,comp}^{e}\left(I_{k}\right)$ Eq (15) are calculated.
6. Finally, the fitness function $E=\sum_{k=1}^{N_{lev}}\sum_{e\in X_{ECAP}}{\left|\phi_{ECAP,comp}^{e}\left(I_{k}\right)-\phi_{ECAP}^{e}\left(I_{k}\right)\right|}^{p}$ is computed. This function evaluates the error between the computed and real ECAPs. A power *p* = 4 has been selected for our applications.

Steps 4 to 6, belonging to the DE algorithm, are iteratively repeated until the stopping criterion (either E < tol or iter > maxiter) is satisfied.

As a result of this algorithm we obtain the parameters *α*, *β* and *Jmax* that minimize the difference between $\phi_{ECAP,com}^{e}\left(I_{k}\right)$ and $\phi_{ECAP}^{e}\left(I_{k}\right)$.

#### Differential evolution algorithm for fitting the clinical data

Differential evolution (DE) is a population based stochastic search technique successfully and widely used as global optimizer inspired by the Darwinian principle of natural selection and genetic reproduction [57, 58]. It is currently being considered as one of the most powerful stochastic real-parameter optimization algorithms [59]. Its application in engineering and applied sciences has contributed to optimize and solve problems in many fields (e.g.: in the bioinformatics and biomedical engineering field, [60]); particularly, in relation with the type of problem required to be solved in this work, it has been applied in parameter estimation (e.g.: [61–63]).

Our optimization problem consists of a chromosome of three variables: *α*, *β* and *J*_*max*_, whose minimum and maximum initial constraint limits for the variables searched are (0, 0, 0) and (2000, 2000, 1500), respectively, although the DE could attain values out of those limits if they improve the fitness function. After trying different DE configurations, we have observed that the problem rapidly converges and the result hardly depends on the selected configuration.

**Fig 10:**
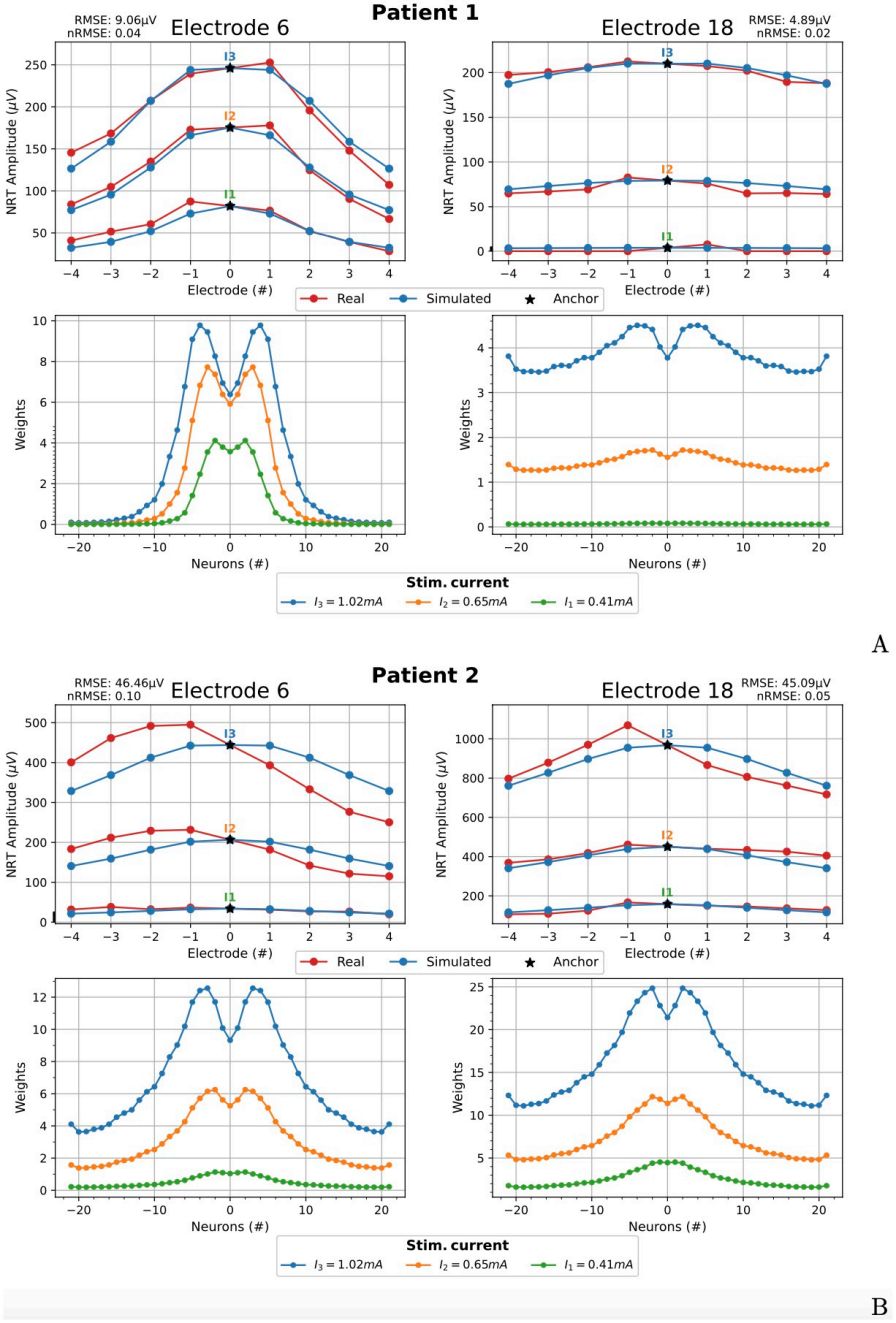
Symmetric fitting. Comparison between real and simulated NRT amplitudes for the two patients at the electrodes 6 (basal) and 18 (apical) (upper row A and B). Corresponding weights ${\bar{w}}_{n}^{0}\left(I_{k}\right)$ (bottom row A and B). The *anchor* electrode is 0 in both cases. RMSE and normalized RMSE (nRMSE) values are shown.

Attained weights used in the Results section were obtained with a population size of 10 individuals, with crossover probability of 1, and stopping criterion set to a maximum of 300 generations (maxiter), or alternatively, achieving a value of the fitness function (as exposed in step 6 of the above section) of tol = 10^−30^. An often used DE/rand/1/bin strategy has been set (as in the aforementioned references of parameter estimation problems: [62, 63]), particularly using per-vector-dither (of F weight). As an example, in Table 1 it is shown the parameters obtained after the optimization for the first test presented in the Results section (Fig 10).

**Table 1:** Parameters obtained after the optimization corresponding to first test of the Results section (Fig 10).

Patient	Stimulating Electrode	*α*	*β*	*J*_*max*_ [A/m^2^]	error (*E*^1/*p*^) [μV]
1	6	4.17	88.35	672.44	25.72
1	18	0.53	3.74	672.44	14.29
2	6	1.13	16.27	672.44	134.27
2	18	0.76	5.43	672.44	140.04

### ECAP recording

The clinical data provided from the Hospital Insular de Gran Canaria correspond to two patients meeting the following selection criteria: adults (> 18 years) implanted with a Cochlear CI532 half-band perimodiolar electrode array [64], having a full insertion of the electrode array, having five or more adjacent active electrodes for the ECAPs recording and being CI holders with more than 6 months of use.

The ECAPs have been recorded by means of the software Custom Sound Evoke Potentials (Cochlear Ltd. Sydney, Australia). The Neural Response Telemetry (NRT) technique records, in a neighboring electrode, the action potential resulted from the stimulus applied on a given electrode. Forward masking [65] was used to reduce the electrical artifact produced by stimulation. From now on, instead of ECAP, the term NRT is used in next sections because it was the procedure used for recording.

The CI532 is numerated from most basal to most apical. The selected stimulating electrodes are 6 (basal) and 18 (apical). The standard NRT measurement typically uses the +2 electrode to record the action potential. A broader set of data is needed in order to adjust the proposed computational model. Therefore, in this work, we have extended the number of recording electrodes until 8 (the 4 consecutive ones on each side).

## Results

We have several options to choose the electrode that interpolates the NRT: the *anchor* electrode. We could either select any of the recording electrodes, or interpolate the value of the NRT in the stimulating electrode (electrode 0), since we do not have NRT measure in this electrode. This last choice has the particularity of producing symmetrical results with respect to the stimulating electrode, that is ${\bar{w}}_{n}^{0}={\bar{w}}_{-n}^{0}$. It is especially suitable when the clinical data present this type of symmetry.

The first test consists in adjusting the NRT values of the two patients using all available data. The *anchor* electrode chosen in this case has been *a* = 0.

Fig 10A (upper row) shows $\phi_{ECAP}^{e}\left(I_{k}\right)$ and the fitted values of $\phi_{ECAP,comp}^{e}\left(I_{k}\right)$ for the three input currents *I*_1_ = 0.41 mA, *I*_2_ = 0.65 mA and *I*_3_ = 1.02 mA for patient 1. The lower part of Fig 10A shows the corresponding weights ${\bar{w}}_{n}^{0}\left(I_{k}\right)$, *n* = {−21, …, 0, …, 21}. Fig 10B shows the same result than A, for patient 2. In general, the results show a good agreement between the real and simulated data. The greatest difference occurs at the electrode 6 of patient 2. This difference is due to the great asymmetry of real data.

**Fig 11:**
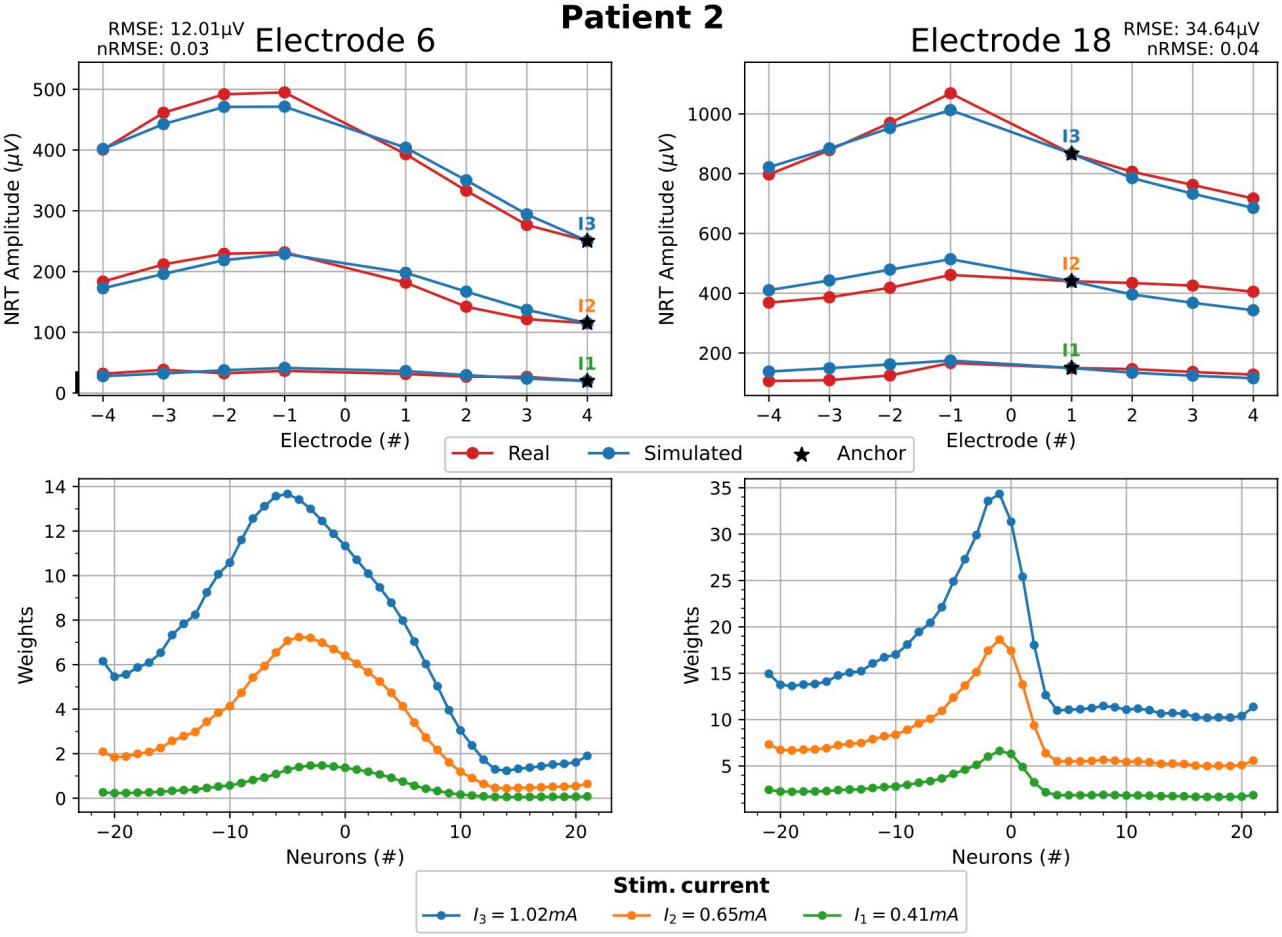
Fitting with optimal anchor. Real and computed NRT amplitudes for the patient 2 choosing the optimum anchor. The global and normalized RMSE of data fitting are shown at the upper row of pictures. Corresponding weights ${\bar{w}}_{n}^{4}\left(I_{k}\right)$ (left) and ${\bar{w}}_{n}^{1}\left(I_{k}\right)$ (right) are shown at the bottom row of pictures.

The selection of the anchor influences the NRT fitting, which is evaluated using the root mean square error (RMSE). The effect of choosing the *optimum anchor*, defined as the one that gives rise to the minimum RMSE, is analyzed here using the real data of Fig 10. Left and right parts of Fig 11 show, for patient 2, the NRTs fitting using electrodes 6 and 18 as the stimulating ones and being electrodes +4 and +1 the optimum anchors, respectively. The optimum anchor for patient 1 is the electrode 0, which is the same as in Fig 10A. So, the results for patient 1 choosing the optimum anchor are not shown in Fig 11.

It can be appreciated that the fitting amplitude of the simulated NRT to the clinical data has improved compared to Fig 10B.

Note that a central anchor imposes a symmetrical fitting for asymmetric data. It is logical that the optimal anchors (+4 and +1) are not the central ones since the clinical data are asymmetric (see Fig 11).

The improvement of the fittings are evaluated using the normalized root mean square errors: nRMSE = RMSE / (max(NRT Amplitude) − min(NRT Amplitude)). In the case of patient 2 and stimulating electrode 6, the nRMSE of the fitting goes from 0.10 (RMSE = 46.46 μV), when we choose the central anchor (Fig 10B, left), to 0.03 (RMSE = 12.01 μV), when using +4 optimal anchor (Fig 11, left), that is, an improvement of 70%. In the case of patient 2 and stimulating electrode 18, the nRMSE of the fitting goes from 0.05 (RMSE = 45.09 μV), when we choose the central anchor (Fig 10B, right), to 0.04 (RMSE = 34.64 μV), when using +1 optimal anchor (Fig 11, right), that is, an improvement of 20%.

**Fig 12:**
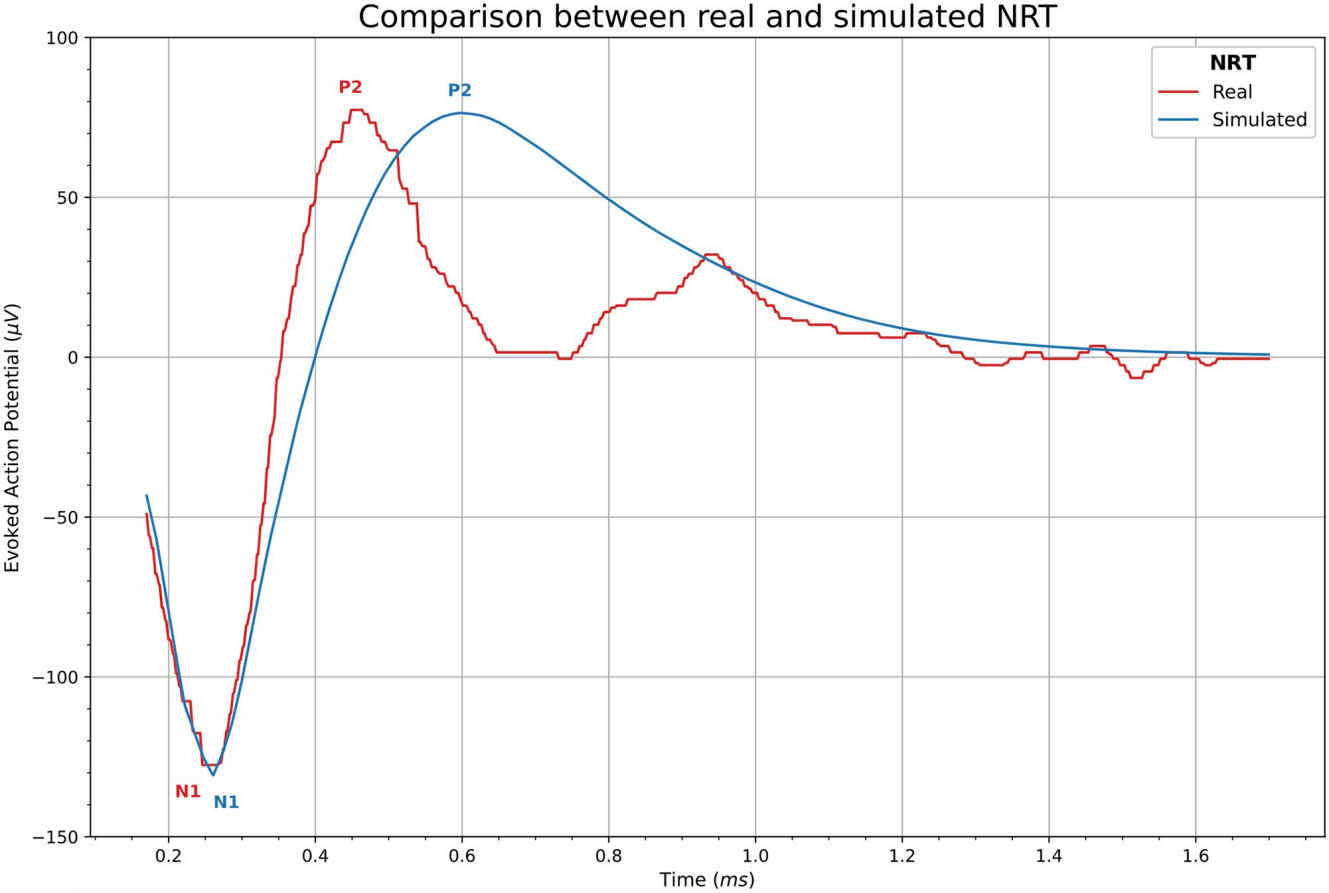
Comparison between real and simulated NRT. Real NRT from patient 1 and stimulating electrode 6 with the intensity *I*_3_ = 1.02 mA and recorded at electrode +2 (red). Computed NRT fitted to the patient data (blue).

In Fig 12 it is shown the real and computed NRTs from the patient 1, after stimulating electrode 6 and recording at electrode 8 (+2 in local numbering). Note that the values of peaks N1 and P2 of the real and computed graphics are similar and so is the time lapse between them.

**Fig 13:**
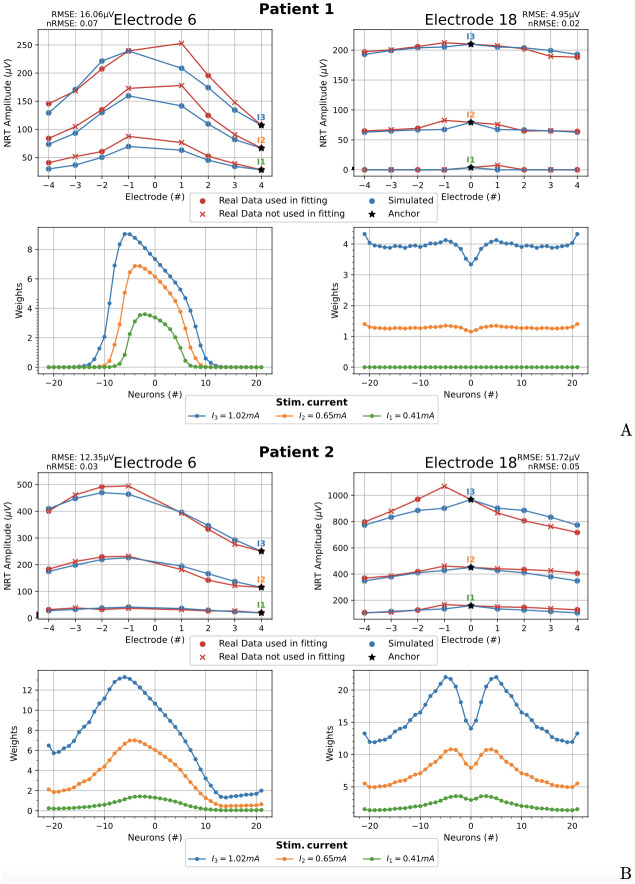
Comparison between real and simulated NRT amplitudes with incomplete training dataset. Resulting NRT amplitudes using only the values from electrodes ±2 and ±4 as training data for the optimization. The *anchor* electrode is the optimal in both cases. RMSE and normalized RMSE (nRMSE) values are shown.

The third test consists in discarding some real data in the fitting procedure of the model. Fig 13 shows the results of $\phi_{ECAP}^{e}\left(I_{k}\right)$ and $\phi_{ECAP,comp}^{e}\left(I_{k}\right)$ after removing the data of the electrodes ±1 and ±3. The *anchor* electrode is the optimum from the available data. It can be seen that, despite the lack of data, the agreement between the real and simulated data is satisfactory.

The last test tries to reproduce the usual situation in which we only know the NRT of the electrode +2 of a certain patient. Lack of data does not allow calculating the *δ*_*n*_ parameters for this patient. A possibility to calculate the weights ${\bar{w}}_{n}^{+2}$ is to take *δ*_*n*_ from other patient and to use the NRT of the patient under study to calculate $w_{n}^{+2}$. Thus, the final weights are ${\bar{w}}_{n}^{+2}=\frac{\delta_{n}}{\Delta }w_{n}^{+2}$.

**Fig 14:**
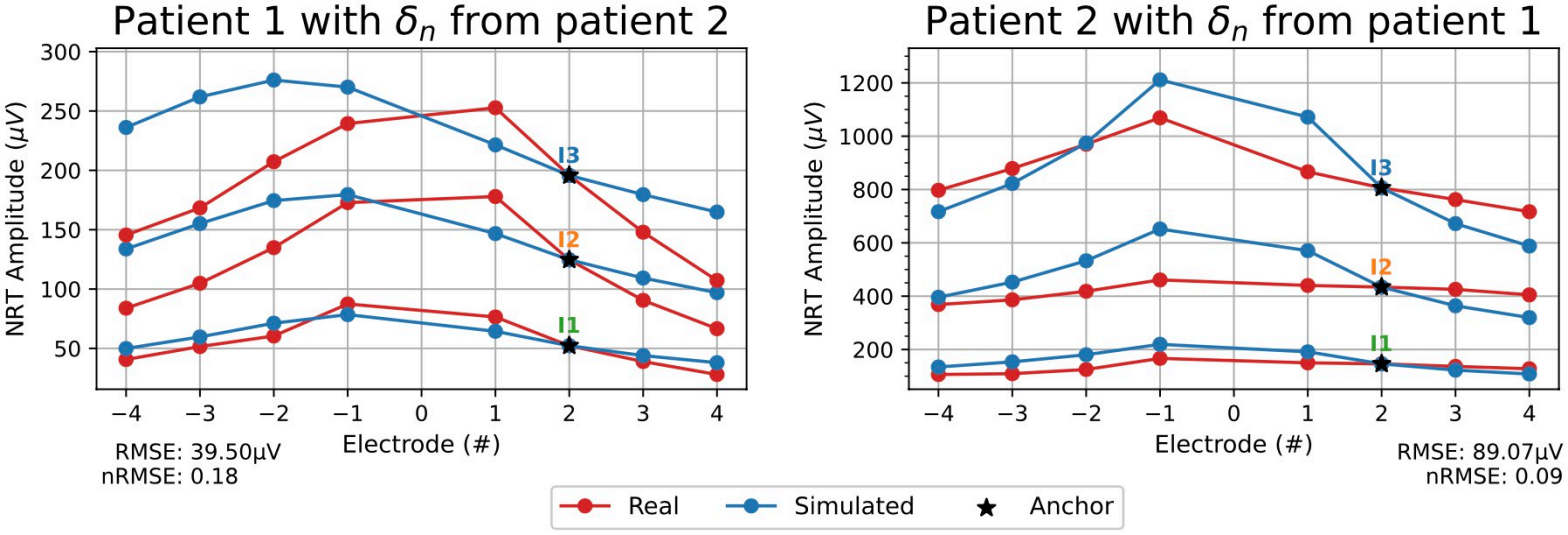
NRT prediction test. NRT amplitudes of the patient 1 and electrode 6 with *δ*_*n*_ taken from the patient 2 (left), and NRT amplitudes of the patient 2 and electrode 18 with *δ*_*n*_ from patient 1 (right). RMSE and normalized RMSE (nRMSE) values are shown. Stimulation currents: *I*_1_ = 0.41 mA, *I*_2_ = 0.65 mA, *I*_3_ = 1.02 mA.

Fig 14 reproduces this situation. In particular, Fig 14 (left) shows the amplitudes of the NRT for the patient 1 and electrode 6 in which the *δ*_*n*_ have been taken from the patient 2. Fig 14 (right) reproduces the same experiment, but now the *δ*_*n*_ of the electrode 18 and patient 1 have been transferred to patient 2. Note that, even in this case, the agreement between real data and computed result is very good.

The RMSE values between clinical and simulated data are shown in Figs 10, 11, 13 and 14.

## Discussion

We have developed a computational model to simulate the neural response to electrical stimulus of CIs. The fitting parameters of our model (the weights) depend on the current density distribution. This dependence gives our model the ability to predict variations in response to changes in the design or configuration of the CI.

One of the most relevant characteristics of our model is its ability to reproduce the NRT values of each patient and electrode. These data can vary widely. For example, the values recorded at electrode 20 (+2 in the local numbering) when we stimulate electrode 18 with a current *I*_3_ = 1.02 mA are 202.18 μV for patient 1 and 806.35 μV for patient 2, as can be seen in the upper right corners of Fig 10A and 10B. That is, the variation in the amplitude of the NRT is 604.17 μV. Our model reproduces the NRTs of each patient with an RMSE less than 46.46 μV (see caption of Fig 10). As far as we know, no other cochlear model published in the literature has this ability to adapt to clinical data.

The example in Fig 14 shows the ability to predict NRT values by simulating the usual situation where only the NRT at the +2 electrode is known. The NRT measurements of the other electrodes have been used only to contrast the model, but not to fit it. In this example, the *δ*_*n*_ parameters of patient 1 are used in patient 2, and vice versa. The greatest discrepancy between the clinical and simulated NRT amplitudes is 205.27 μV, that is, 23.7%, and it occurs in patient 2 and electrode 1 with a current *I*_3_ = 1.02 mA (Fig 14 right). This experiment shows, to some extent, the predictive capacity of our model. Nevertheless, it would be necessary to collect data from more patients to validate such predictive capacity. We will intend to complete a larger database in the future.

The model could help in the classification of patients according to the type of pathology. For example, it could be useful to determine dead regions of the auditory nerve. Our model can also be used to determine the focusing capacity of an electrode [12]. A higher focusing capacity is linked to a lower spreading of the current and, therefore, a higher concentration of the weights around the stimulating electrode.

Our straight cochlea model has certain limitations. For instance, it produces symmetric transimpedances when the central electrode is stimulated. The symmetry of the model implies that *Z*_*i*0_ = *Z*_−*i*0_. This means that the potential registered at the electrode *i* is equal to the potential registered in the electrode −*i*, when the electrode 0 (central) is stimulated. The construction of a more realistic 3D geometric model could improve the concordance between the clinical data and its modeling. Furthermore, a patient-adapted model can be built from computerized tomography scans [66].

The conductivity *σ*_*ext*_ has been fitted using the transimpedances of patient one, but the model could be fitted by using the specific values of each patient.

Our model considers that all the VN are activated at the same time. The biological reality is more complex and there is not a perfect synchronization in the activation of neurons. This lack of synchronization may have some effect on the recorded ECAP and could be taken into account in future work.

In this work we have considered that the propagation velocity of the action potential is uniform throughout the neuron. However, this velocity is different depending on the section of the neuron considered, due to the change in the diameters between axon and dendrite [53]. This question could be considered in future work.

The input membrane current used in this work was provided by [35]. However, another H-H-type model could be taken for providing the input current, although the weights obtained after adjusting the computed ECAP to the clinical data would be different, given that the amplitude of the membrane current could vary according to the type of H-H model selected.

In this study only ionic and leak currents were used. This is because clinical records, obtained by the NRT technique, only present the N1-P2 response due to the electrical artifact cancellation procedure (see Fig 12). If we use a multi-compartment model it is possible to include the capacitive current, but in this case appears the P1-N1-P2 complex. If we wanted to include the capacitive current, we would have to remove the part of the wave that contains P1 in order to adjust the computed ECAP to the real recording.

Our phenomenological model, by construction, will always find the optimal solution to adjust the ECAPs. As indicated in Method section, the weights represent the number of activated neurons that each virtual neuron represents. The interpretation of the weights will be more or less realistic depending on the data provided to the model (more realistic geometry, conductivities, density of the auditory nerve, etc.). The more the model conforms to reality, the more credible the interpretation of the weights will be.

## References

[1] Prochazka A. Neurophisiology and neural engineering: a review. Journal of Neurophysiology, 2017, Vol. 118: pp. 1292–1309. doi: 10.1152/jn.00149.2017 PMC5558026 28566462

[2] Lenarz T. Cochlear Implant—State of the Art, GMS Current Topics in Otorhinolaryngology. Head and Neck Surgery, 2017, Vol. 16, pp. 1–29 10.3205/cto000143 PMC5818683 29503669

[3] de Vos JJ, Biesheuvel JD, Briaire JJ, Boot PS, van Gendt MJ, Dekkers OM, Fiocco M. Frijns JHM. Use of Electrically Evoked Compound Action Potentials for Cochlear Implant Fitting: A Systematic Review. Ear and Hearing, 2017, vol. 39-3, pp. 401–411. 10.1097/AUD.0000000000000495 28945656

[4] Westen AA, Dekker DMT, Briaire JJ, Frijns JHM. Stimulus level effects on neural excitation and eCAP amplitude. Hear. Res. 280, 166–176, 2011. doi: 10.1016/j.heares.2011.05.014 21664959

[5] He S, Teagle HFB, Buchman CA, The Electrically Evoked Compound Action Potential: From Laboratory to Clinic. Front. Neurosci., Vol. 11, Article 339. 2017 10.3389/fnins.2017.00339 PMC5481377 28690494

[6] Mangado N, Ceresa M, Benav H, Mistrik P, Piella G, Ballester MAG. Towards a Complete In Silico Assessment of the Outcome of Cochlear Implantation Surgery. Molecular Neurobiology. 2017;55(1):173–186. doi: 10.1007/s12035-017-0731-z 28840488

[7] Whiten DM. Electro-anatomical models of the cochlear implant. PhD Thesis, Massachusetts Institute of Technology, 2007. Available from: https://dspace.mit.edu/handle/1721.1/38518.

[8] Dang K. Electrical conduction models for cochlear implant stimulation. Other. Université Côte d’Azur, 2017. English. NNT: 2017AZUR4043. tel-01562277v2, 2017. Available from: https://hal.inria.fr/tel-01562277v2.

[9] Joucla S, Yvert B. Improved Focalization of Electrical Microstimulation Using Microelectrode Arrays: A Modeling Study. PLoS ONE. 2009;4(3):e4828. doi: 10.1371/journal.pone.0004828 19279677 PMC2652101

[10] Kalkman RK, Briaire JJ, Frijns JHM. Current focussing in cochlear implants: An analysis of neural recruitment in a computational model. Hearing Research. 2015;322:89–98. doi: 10.1016/j.heares.2014.12.004 25528491

[11] Xu Y, Luo C, You Z. Optimization of cochlear Implants stimulation resolution using an intracochlear electric potential model. Computers in Biology and Medicine. 2018;94:99–105. doi: 10.1016/j.compbiomed.2017.12.016 29408002

[12] de Miguel ÁR, Escobar JM, Greiner D, Ramos-Macías Á. A multiobjective optimization procedure for the electrode design of cochlear implants. International Journal for Numerical Methods in Biomedical Engineering. 2018;34(8):e2992. doi: 10.1002/cnm.2992 29633585

[13] Zhang T, Dorman MF, Gifford R, Moore BCJ. Cochlear Dead Regions Constrain the Benefit of Combining Acoustic Stimulation With Electric Stimulation. Ear & Hearing. 2014;35(4):410–417. doi: 10.1097/AUD.0000000000000032 24950254 PMC4066196

[14] van Gendt MJ, Briaire JJ, Frijns JHM. Effect of neural adaptation and degeneration on pulse-train ECAPs: A model study. Hear. Res. 377, 167–178, 2019. doi: 10.1016/j.heares.2019.03.013 30947041

[15] Bruce IC, White MW, Irlicht LS, O’Leary SJ, Dynes S, Javel E, Graeme M, Clark A. A Stochastic Model of the Electrically Stimulated Auditory Nerve: Single-Pulse Response IEEE Transactions on Biomedical Engineering, Vol. 46, No. 6, June 1999 doi: 10.1109/10.764939 10356868

[16] Briaire JJ, Frijns JHM. Unraveling the electrically evoked compound action potential. Hear. Res. 205, 143–156, 2005. doi: 10.1016/j.heares.2005.03.020 15953524

[17] Rattay F, Danner S. M. Peak I of the human auditory brainstem response results from the somatic regions of type I spiral ganglion cells: Evidence from computer modeling. Hear. Res. 315, 67–79, 2014. doi: 10.1016/j.heares.2014.07.001 25019355 PMC4152002

[18] Ceresa M, Mangado N, Andrews RJ, Ballester MAG. Computational Models for Predicting Outcomes of Neuroprosthesis Implantation: the Case of cochlear implants. Molecular Neurobiology. 2015;52(2):934–941. doi: 10.1007/s12035-015-9257-4 26084438

[19] Malherbe TK, Hanekom T, Hanekom JJ. The effect of the resistive properties of bone on neural excitation and electric fields in cochlear implant models. Hearing Research. 2015;327:126–135. doi: 10.1016/j.heares.2015.06.003 26074305

[20] Malherbe TK, Hanekom T, Hanekom JJ. Constructing a three-dimensional electrical model of a living cochlear implant user’s cochlea. International Journal for Numerical Methods in Biomedical Engineering. 2015;32(7):e02751. doi: 10.1002/cnm.2751 26430919

[21] Mangado N, Pons-Prats J, Coma M, Mistrík P, Piella G, Ceresa M, et al. Computational Evaluation of cochlear implant Surgery Outcomes Accounting for Uncertainty and Parameter Variability. Frontiers in Physiology. 2018;9. doi: 10.3389/fphys.2018.00498 29875673 PMC5975103

[22] Choi CTM, Wang SP. Modeling ECAP in Cochlear Implants Using the FEM and Equivalent Circuits. IEEE Trans. Magn. 50, 49–52, 2014. doi: 10.1109/TMAG.2013.2282640

[23] Briaire JJ, Frijns JHM. The consequences of neural degeneration regarding optimal cochlear implant position in scala tympani: A model approach. Hear. Res. 214, 17–27, 2006. doi: 10.1016/j.heares.2006.01.015 16520009

[24] Potrusil T, Heshmat A, Sajedi S, Wenger C, Johnson Chacko L, Glueckert R, Schrott-Fischer A, Rattay F. Finite element analysis and three-dimensional reconstruction of tonotopically aligned human auditory fiber pathways: A computational environment for modeling electrical stimulation by a cochlear implant based on micro-CT. Hear Res., Vol. 393, pp. 108001, 2020. doi: 10.1016/j.heares.2020.108001 32535276

[25] Nogueira W, Schurzig D, Büchner A, Penninger RT, Würfel W. Validation of a cochlear implant Patient-Specific Model of the Voltage Distribution in a Clinical Setting. Frontiers in Bioengineering and Biotechnology. 2016;4. doi: 10.3389/fbioe.2016.00084 27933290 PMC5120131

[26] Hanekom T, Hanekom JJ. Three-dimensional models of cochlear implants: A review of their development and how they could support management and maintenance of cochlear implant performance. Network: Computation in Neural Systems. 2016;27(2-3):67–106. doi: 10.3109/0954898X.2016.1171411 27136100

[27] Kalkman RK, Briaire JJ, Dekker DM, Frijns JH. Place pitch versus electrode location in a realistic computational model of the implanted human cochlea. Hear Res. 315:10–24, 2014. doi: 10.1016/j.heares.2014.06.003 24975087

[28] Nogueira W, Litvak LM, Landsberger DM, Büchner A. Loudness and pitch perception using Dynamically Compensated Virtual Channels. Hear Res., Vol.344, pp.223–234. doi: 10.1016/j.heares.2016.11.017 27939418 PMC5421637

[29] Jürgens T, Hohmann V, Büchner A, Nogueira W. The effects of electrical field spatial spread and some cognitive factors on speech-in-noise performance of individual cochlear implant users—A computer model study. PLoS ONE 13(4): e0193842, 2018. doi: 10.1371/journal.pone.0193842 29652892 PMC5898708

[30] Nogueira W, Ashida G. Development of a Parametric Model of the Electrically Stimulated Auditory Nerve. In: Biomedical Technology: Modeling, Experiments and Simulation, Eds.: Wriggers P and Lenarz T, Springer International Publishing, 2018, 349–362. doi: 10.1007/978-3-319-59548-1_19

[31] Dong Y, Briaire JJ, Biesheuvel JD, Stronks HC, Frijns JHM. Unravelling the temporal properties of human eCAPs through an iterative deconvolution model. Hear Res., Vol. 395, 2020. doi: 10.1016/j.heares.2020.108037 32827881

[32] Bai S, Encke J, Obando-Leitón M, Weiß R, Schäfer F, Eberharter J, Böhnke F, Hemmert W. Electrical stimulation in the human cochlea: A computational study based on high-resolution micro-CT scans. Frontiers in neuroscience, Vol. 13, 1312, 2019. doi: 10.3389/fnins.2019.01312 31920482 PMC6915103

[33] Hodgkin AL, Huxley AF. A quantitative description of membrane current and its application to conduction and excitation in nerve. J Physiol. 117(4):500–544, 1952. doi: 10.1113/jphysiol.1952.sp004764 12991237 PMC1392413

[34] Ermentrout GB, Terman DH. Mathematical Foundations of Neuroscience. Springer New York; 2010. Available from: 10.1007/978-0-387-87708-2.

[35] Ashida G, Nogueira W. Spike-Conducting Integrate-and-Fire Model. eneuro. 2018;5(4):ENEURO.0112–18.2018. doi: 10.1523/ENEURO.0112-18.2018 30225348 PMC6140110

[36] de Miguel ÁR, Durmo I, González JCF, Barreiro SB, Macías AR. Evaluation of Intracochlear Position of a Slim Modiolar Electrode Array, by Using Different Radiological Analyses. Otology & Neurotology. 2019;40(5S):S10–S17. doi: 10.1097/MAO.0000000000002213 31225817

[37] Saba R. Cochlear implant modelling: stimulation and power consumption [PhD dissertation]. Faculty of Engineering and the Environment. University of Southampton; 2012. Available from: https://eprints.soton.ac.uk/.

[38] Kumar G, Chokshi M, Richter C. Electrical impedance measurements of cochlear structures using the four-electrode reflection-coefficient technique. Hear Res., Vol. 259, Issues 1–2, pp. 86–94, 2010. doi: 10.1016/j.heares.2009.10.010 19857561

[39] Moller AR, Colletti V, Fiorino G. Neural conduction velocity of the human auditory nerve: bipolar recordings from the exposed intracranial portion of the eighth nerve during vestibular nerve section. Electroencephalography and clinical Neurophysiology. 1994;92:316–320. doi: 10.1016/0168-5597(94)90099-X 7517853

[40] Bachmaier R, Encke J, Obando-Leitón M, Hemmert W, Bai S. Comparison of Multi-Compartment Cable Models of Human Auditory Nerve Fibers. Frontiers in Neuroscience. 2019;13. doi: 10.3389/fnins.2019.01173 31749676 PMC6848226

[41] Joucla S, Yvert B. Modeling extracellular electrical neural stimulation: From basic understanding to MEA-based applications. Journal of physiology, Paris. 106. 146–158, 2012. doi: 10.1016/j.jphysparis.2011.10.003 22036892

[42] Haus JRM Hermann A. Electromagnetic Fields and Energy. Englewood Cliffs, NJ: Massachusetts Institute of Technology: MIT OpenCourseWare; 1989.

[43] Chu Y, Cao Y, He X, Luo M. Asymptotic boundary conditions with immersed finite elements for interface magnetostatic/electrostatic field problems with open boundary. Computer Physics Communications. 2011;182(11):2331–2338. doi: 10.1016/j.cpc.2011.06.014

[44] Gratkowski S. General Closed-Form Asymptotic Boundary Conditions for Finite Element Analysis of Exterior Electrical Field Problems. Przeglad Elektrotechniczny. 2016;1(5):15–18. doi: 10.15199/48.2016.05.04

[45] Holt GR. A critical reexamination of some assumptions and implications of cable theory in neurobiology [PhD dissertation]. California Institute of Technology. Pasadena, California; 1998. Available from: https://thesis.library.caltech.edu/3499/.

[46] Holt GR, Koch C. Electrical Interactions via the Extracellular Potential Near Cell Bodies. Journal of Computational Neuroscience. 1999;6(2):169–184. doi: 10.1023/A:1008832702585 10333161

[47] Pods J, Schönke J, Bastian P. Electrodiffusion Models of Neurons and Extracellular Space Using the Poisson-Nernst-Planck Equations—Numerical Simulation of the Intra- and Extracellular Potential for an Axon Model. Biophysical Journal. 2013;105(1):242–254. doi: 10.1016/j.bpj.2013.05.041 23823244 PMC3703912

[48] Parasuram H, Nair B, D'Angelo E, Hines M, Naldi G, Diwakar S. Computational Modeling of Single Neuron Extracellular Electric Potentials and Network Local Field Potentials using LFPsim. Frontiers in Computational Neuroscience. 2016;10. doi: 10.3389/fncom.2016.00065 27445781 PMC4923190

[49] Pods J. A comparison of computational models for the extracellular potential of neurons. Journal of Integrative Neuroscience. 2017;16(1):19–32. doi: 10.3233/JIN-170009 28891501

[50] Pods J.J. Electrodiffusion Models of Axon and Extracellular Space Using the Poisson-Nernst-Planck Equations [PhD dissertation]. Heidelberg University Library; 2014.

[51] Wang XJ, Buzsáki G. Gamma oscillation by synaptic inhibition in a hippocampal interneuronal network model. J. Neurosci. 16:6402–13, 1996. doi: 10.1523/JNEUROSCI.16-20-06402.1996 8815919 PMC6578902

[52] Ghanaei A, Firoozabadi SM, Sadjedi H. A fast approximate method for predicting the behavior of auditory nerve fibers and the evoked compound action potential (ECAP) signal. J. Med. Signals Sensors 11, 169, 2021. doi: 10.4103/jmss.JMSS_28_20 34466396 PMC8382029

[53] Rattay F, Potrusil T, Wenger C, Wise AK, Glueckert R, Schrott-Fischer A Impact of Morphometry, Myelinization and Synaptic Current Strength on Spike Conduction in Human and Cat Spiral Ganglion Neurons. PLOS ONE 8(11): e79256, 2013 doi: 10.1371/journal.pone.0079256 24260179 PMC3832640

[54] Joucla S, Glière A, Yvert B. Current approaches to model extracellular electrical neural microstimulation. Frontiers in Computational Neuroscience. 2014;8. doi: 10.3389/fncom.2014.00013 24600381 PMC3928616

[55] Rotem A, Moses E. Magnetic Stimulation of One-Dimensional Neuronal Cultures. Biophysical Journal. 2008;94(12):5065–5078. doi: 10.1529/biophysj.107.125708 18326634 PMC2397342

[56] Devore JL. Probability and Statistics for Engineering and the sciences. California, San Luis Obispo: CENGAGE Learning; 2016.

[57] Storn R, Price K. Differential evolution–a simple and efficient heuristic for global optimization over continuous spaces. Journal of global optimization. 1997;11(4):341–359. doi: 10.1023/A:1008202821328

[58] Price K, Storn RM, Lampinen JA. Differential evolution: a practical approach to global optimization. Springer Science & Business Media; 2006.

[59] Das S, Mullick S.S, Suganthan P.N. Recent advances in differential evolution–an updated survey. Swarm and Evolutionary Computation. 2016;27:1–30. doi: 10.1016/j.swevo.2016.01.004

[60] Mesejo P, Ugolotti R, Di Cunto F, Giacobini M, Cagnoni S. Automatic hippocampus localization in histological images using differential evolution-based deformable models. Pattern Recognition Letters. 2013;34(3):299–307. doi: 10.1016/j.patrec.2012.10.012

[61] González F., Greiner D, Mena V, Souto R.M, Santana J.J., Aznárez J.J. Fitting procedure based on Differential Evolution to evaluate impedance parameters of metal–coating systems. Engineering Computations. 2019;36(9):2960–2982. doi: 10.1108/EC-11-2018-0513

[62] Tang Y, Zhang X, Hua C, Li L, Yang Y. Parameter identification of commensurate fractional-order chaotic system via differential evolution. Physics Letters A. 2012;376(4):457–464. doi: 10.1016/j.physleta.2011.12.008

[63] Zhan C, Situ W, Yeung LF, Tsang PWM, Yang G. A parameter estimation method for biological systems modelled by ODE/DDE models using spline approximation and differential evolution algorithm. IEEE/ACM transactions on computational biology and bioinformatics. 2014;11(6):1066–1076. doi: 10.1109/TCBB.2014.2322360 26357044

[64] Aschendorff A, Briggs R, Brademann G, Helbig S, Hornung J, Lenarz T, et al. Clinical investigation of the Nucleus Slim Modiolar Electrode. Audiology and Neurotology. 2017;22(3):169–179. doi: 10.1159/000480345 29059669

[65] Shannon RV. Forward masking in patients with cochlear implants. The Journal of the Acoustical Society of America. 1990;88(2):741–744. doi: 10.1121/1.399777 2212298

[66] Mangado N, Ceresa M, Duchateau N, Kjer HM, Vera S, Velardo HD, et al. Automatic Model Generation Framework for Computational Simulation of cochlear implantation. Annals of Biomedical Engineering. 2015;44(8):2453–2463. doi: 10.1007/s10439-015-1541-y 26715210

